# Supporting Parents to Support Children: A U.K. Randomized Controlled Trial Testing a Text Message Intervention to Cultivate the Home Learning Environment

**DOI:** 10.1037/edu0000973

**Published:** 2025-08-28

**Authors:** Fionnuala O’Reilly, Susanna Loeb, Alice Farrell, Gaia Scerif

**Affiliations:** 1 Department of Experimental Psychology, University of Oxford; 2 Stanford Graduate School of Education; 3 Department for Energy Security and Net Zero, HM Government, London, United Kingdom

**Keywords:** parents, text message, home learning environment, language, socioemotional skills

## Abstract

A stimulating and supportive home learning environment helps children to be cognitively and emotionally ready to learn by age 5. Despite the growing availability of parenting resources, the attainment gap between affluent and disadvantaged families continues to widen across many developed nations. Tips by Text is a 12-month text message program originally developed in the United States to help parents integrate developmental activities into daily routines with their children. Designed for 4- to 5-year-olds, the messages aim to support language, literacy, numeracy, and socioemotional skills. We adapted and tested the Tips by Text program in a large-scale randomized controlled trial involving 109 schools in England from 2019 to 2021 (*n* = 3,649). Due to COVID-19, postintervention data collection faced approximately 70% attrition on the primary outcome measure, resulting in a final analytical sample of *n* = 753. Despite this, the retained sample was comparable to the original U.S. study, allowing us to proceed with analysis. In this study, we replicate the primary impact analysis conducted by the original randomized controlled trial evaluators and extend it to examine whether the intervention had differential effects on children facing various challenges, including socioeconomic disadvantage, language barriers, special educational needs, or multiple overlapping risk factors. Our analysis found no significant improvements in reading-related skills or socioemotional outcomes nor any meaningful differences in response to the program based on these subgroups. The high level of attrition limited the study’s power to detect effects, underscoring the need for further evaluation, which is currently underway.

## Educational Impact and Implications Statement

The home learning environment plays a crucial role in shaping early development and may help mitigate disparities between children from disadvantaged backgrounds and their more advantaged peers. Through a large-scale randomized controlled trial, we examined whether sending parents of 4- to 5-year-olds short, actionable tips via short message service could enhance their children’s reading-related skills and socioemotional well-being. This article presents secondary data analyses exploring the program’s impact on students facing additional challenges, including socioeconomic disadvantage, English as an additional language, and special educational needs. The intervention did not lead to significant improvements in reading-related skills or socioemotional outcomes, including for children from disadvantaged backgrounds, English as an additional language learners, or those with special educational needs. However, high attrition because of the COVID-19 pandemic reduced the study’s power to detect effects. Further evaluation is needed and is already underway. This research contributes to our understanding of whether and how simple, regular prompts can effectively support parental engagement in early learning.

Researchers and practitioners increasingly acknowledge that experiences in the first 5 years of life have significant, lasting effects on a child’s development into adulthood (Britto et al., 2017). Early skills form the basis for more complex abilities (Entwisle et al., 2005; Kautz et al., 2014). A child’s traits, knowledge, and skills interact with their environment, shaping their development through feedback (Meisels, 1998). This dynamic interaction critically influences their growth trajectory (Bronfenbrenner & Ceci, 1994; Bronfenbrenner & Morris, 1998). Early skills are highly adaptable, and parents play a critical role as primary influencers in their child’s life. This study replicates and extends analyses of a text message intervention, Tips by Text, which aims to support parents in creating enriching home environments that foster positive developmental outcomes. In the original analysis conducted by the appointed evaluators, no significant impact was observed on either the primary or secondary outcome measures, with a 70% attrition rate recorded for the primary outcome because of the COVID-19 pandemic. Here, we extend the investigation to focus on specific subgroups, including children facing various forms of socioeconomic challenge, language barriers, and SEN, as well as examining the combined effect of these challenges.

According to the United Nations, around one-third of children aged 3–4 years in 72 different countries fail to achieve the expected developmental milestones in literacy and numeracy (United Nations, 2019). In England, less than half (48.1%) of children eligible for free school meals (FSMs)—an indicator of socioeconomic disadvantage—achieved the required “good level of development” on the Early Years Foundation Stage Profile (EYFSP) in 2022. In the same year, the attainment gap between FSM and non-FSM children stood at 19.6 percentage points (Orso & Wilcock, 2022). This disparity underscores the need to understand and ameliorate this significant gap from as early as possible for low-income children. An enriching home learning environment could be a significant protective factor contributing to mitigating this disparity (E. C. Melhuish et al., 2008).

## The Home Learning Environment

A safe, stimulating, and supportive home is crucial for children’s physical, cognitive, and emotional development (Toth et al., 2020). Activities such as shared book reading, the use of complex language, scaffolding, and warm, responsive interactions are all associated with better developmental outcomes by the time children start school (Bornstein et al., 2008; Bradley & Corwyn, 2005; Hart et al., 1997). The presence of materials like books and toys, along with the quality of implicit and explicit learning support from parents and caregivers, fosters effective home learning environments (E. Melhuish, 2010).

The linkages between the home learning environment and emergent academic skills are well established (Lehrl et al., 2020), although much debate exists on how to best measure the quantity and quality of enriching activities in the home, both cross-culturally and in diverse settings within countries (Hornburg et al., 2021). Enriching home environments are associated with improved early reading (Niklas & Schneider, 2013; Sénéchal & LeFevre, 2002) and math skills (Mutaf-Yıldız et al., 2020; Niklas & Schneider, 2013; Skwarchuk et al., 2014). These relations remain stable into early adolescence, and the developmental pathways are observed across different ethnic and racial groups (Tamis-LeMonda et al., 2017).

Like quality metrics for early years educational settings, the quality of the home environment can be broken down into structural and process factors (Anders et al., 2011; Foster et al., 2005). Structural characteristics include income, housing, and parental education. Process factors relate to the nature of interactions between the child and parents, other children, and the child’s engagement with their physical surroundings and resources within the home. Parental views of technology and digital resources for learning are now also an important component of the home environment (Cahoon et al., 2024).

These structural and process characteristics of the home environment are situated within a wider context, as described by Bronfenbrenner’s (1986) bioecological systems theory. This theory encompasses the interconnected forces that shape a child, acknowledging both distal factors like cultural norms and proximal influences, primarily parents and the home environment (Bronfenbrenner, 1986). Parenting is challenging under any circumstances, but it becomes even more difficult when parents lack the necessary support structures to create stimulating and supportive learning environments. Deficit-based accounts that highlight parental shortcomings in the context of poverty overlook the resilience and determination of these families (DeJoseph et al., 2024; Ellis et al., 2017). In England, the past decade has seen an erosion of such services for families with young children. The closure of children’s centers (Carneiro et al., 2024)^1^ and local libraries (Chartered Institute of Public Finance and Accountancy, 2023) has meant that parents have less access to reliable parenting advice and local support networks. Despite this, evidence from the Millennium Cohort Study in the United Kingdom suggests that mothers from low-income households are finding creative, low-cost, and enriching activities to engage their children (Cooper, 2021). In the face of structural cuts, devising other approaches to support families is necessary. Programs delivered in collaboration with schools, which offer support to parents, may be one such approach.

## Interventions to Improve the Home Learning Environment

Extensive research has evaluated the effectiveness of various parenting programs. These programs encompass individual and group-based training sessions delivered both online and offline, including apps and text message initiatives. The primary goal of these interventions is to equip parents with the skills needed to support their children’s cognitive, socioemotional, and physical development.

Several meta-analyses, such as those by Mol et al. (2008) and Sénéchal and Young (2008), have highlighted the potential of home learning interventions to enhance child competencies (Mol et al., 2008; Sénéchal & Young, 2008). More recent systematic reviews and meta-analyses (see Gao et al., 2020; Jeong et al., 2021; Wittkowski et al., 2016) of programs targeting parents of children under 6 years report small to medium effect sizes on various child outcomes including cognitive development, language skills, motor skills, socioemotional development, and attachment. Additionally, these studies find positive effects on parent knowledge and behavior. These findings are encouraging, but many such programs require parents’ direct involvement in face-to-face sessions, a constraint that may be out of reach for parents experiencing multiple access barriers (e.g., irregular work schedules, lack of transport, and English as an additional language [EAL]). Less intensive programs that offer parents accessible information and straightforward strategies without requiring a fixed time commitment present a promising alternative, potentially engaging parents who might not participate in more structured programs.

### Text Messages: A Promising Approach to Boost Parental Engagement

Recent years have seen a proliferation of apps for parents available on iOS and Android. As of the end of 2021, the Google Play Store hosted 7,819 separate apps on its platform, all targeting parents of children aged 0–18 (Fedorychak, 2024). While apps offer several advantages, such as the potential for engaging content through branding and gamification, they also present significant drawbacks. One major challenge is convincing parents to download an app and use it consistently. Additionally, if parents disable push notifications, the ability to provide regular content—crucial to many interventions of this type—is immediately compromised. Another issue is the persistent “digital divide” in parents’ ability to use various technologies, including apps. Higher socioeconomic status (SES) parents and children are more digitally advantaged, which can lead to some parents, who stand to benefit the most from additional support, missing out (Zhang & Livingstone, 2019).

In contrast, text messages provide one of the simplest and most effective methods for delivering content. The 160-character limit encourages developers to be concise, ensuring that only key information is conveyed. Partnering with schools to deliver content via text message can enhance effectiveness, as parents tend to pay close attention to messages from their child’s school, especially when their child is starting formal education (i.e., reception). Most schools in England already use texting platforms for logistical purposes, such as reminders about sports gear or school trips. The Tips by Text program repurposes these platforms, using them to support parents in fostering their children’s cognitive and socioemotional development.

#### Existing Evidence

There is growing evidence to suggest that text messages may be an effective way to engage parents in positive behaviors. In older children and adolescents, text message interventions have been shown to increase attendance rates (Bergman & Chan, 2017), the number of students passing summer school (Kraft & Rogers, 2015), applications for financial aid (Page et al., 2020), and college enrolments (Avery et al., 2021).

In the preschool context, text messages have contributed to reduced chronic absenteeism (Smythe-Leistico & Page, 2018) and increased applications for childcare subsidies (Weixler et al., 2020). There is also growing evidence that text messages can help improve academic skills. For example, Doss et al. (2022) found that a blended program (literacy, math, and socioemotional learning content) improves early math skills among girls (*SD* = 0.156, *p* = .16; Doss et al., 2022). Similarly, Cabell et al. (2019) found that children with more advanced initial language and literacy skills benefit from a text message program designed to promote these skills, while those with lower initial skill levels do not improve in language but instead benefit from the control messages focusing on health and well-being (Cabell et al., 2019). This finding highlights the importance of understanding and tailoring such interventions to the specific needs of individual families. Some families may require support in foundational areas like sleep, nutrition, health, and behavior before they can fully benefit from more academic content. Finally, Bigelow et al. (2020) investigated whether supplementing a home visiting program with text messages improves parental engagement and fidelity (Bigelow et al., 2020). While the researchers found no evidence of overall improvement in child language outcomes, the text messages did result in better adherence to the program content.

Oftentimes in studies testing text message interventions, the messages are sent by researchers which can later prove problematic when attempting scale up, which generally requires delivery to be handled by nursery or school staff. Snell et al. (2022) developed a 5-month text message intervention, “Text to Talk,” where teachers sent weekly vocabulary-building activities to parents, aligned with class content. They hypothesized that teacher involvement would strengthen the parent–teacher relationship. The results indicated that children in the intervention group learned significantly more target words than those in the control group (*d* = 0.17, *p* < .05), highlighting the potential importance of teacher participation in such programs (Snell et al., 2022).

In summary, there is now sufficient research evidence to indicate that low-intensity interventions in parenting and educational fields may have important effects (Mayer et al., 2019). Such interventions are commonly designed to address issues of social inequalities and are planned to do so with low-cost delivery. However, these “nudge” interventions (behavioral economics terminology) will have minimal impact when there are larger adverse structural conditions (e.g., extensive neighborhood poverty and poorly resourced schools).

#### Tips by Text

In this study, we tested the efficacy of a preschool program called “Tips by Text”; a 12-month text messaging intervention for parents of reception students (age 4–5 years)^2^ that aims to break down behavioral barriers to parenting by giving parents concise, actionable tips on a regular basis. Each week, parents received three text messages with short descriptions of simple activities to do at home with their child. The activities targeted language, literacy, socioemotional, and numeracy skills.

The first message labeled, “FACT,” provided parents with information about the specific skill being targeted that week and explained its importance in child development. This message sought to bridge any gaps in parents’ knowledge and understanding. The second message, a “TIP” offered parents a simple, practical activity to incorporate into their daily routines using common household objects. By breaking down the skill into manageable tasks, this message aimed to reduce the cognitive load for parents. The final message each week, the “GROWTH” message, reinforced the long-term benefits of consistently engaging in these activities with children. The program focused on enhancing the quality of learning interactions between parents and children, supporting parents to leverage everyday routines and household objects to create enriching learning experiences. See SI 1 in the online supplemental materials for a sample of the text messages and SI 2 in the online supplemental materials for the theory of change.

Tips by Text was first developed and tested via a randomized controlled trial (RCT; *n* = 821) in the United States and showed a positive impact of approximately 0.11 *SD* on early reading-related skills among 4-year-olds, as measured by the Phonological Awareness Literacy Screening (PALS). Children who scored below the median of the baseline skills distribution disproportionately benefited, showing a 0.31 *SD* increase in scores. This result suggests that Tips by Text could help to reduce achievement gaps if rolled out more widely (York et al., 2019). In a more recent RCT, also in the United States, 3- to 4-year-olds (*n* = 381) from low-income backgrounds and who were not attending preschool showed improvements on the same measure, PALS (treatment effect = 0.209, *p* = .016), controlling for preintervention scores, child age, and caregiver language (Chamberlain et al., 2021).

## Understanding the Effectiveness of Interventions for Different Groups

Average treatment effects can mask important differences in how specific subgroups respond to an intervention, potentially overlooking variations in efficacy and overall benefit for diverse populations (Dahabreh et al., 2016). In this study, we examine the impact of the Tips by Text intervention on three subgroups; those from different socioeconomic groups, those who speak EAL, and those who have SEN. We also explore if the program has a differential impact on students who experience more than one of these challenges.

### SES

The disparities in parenting practices between economically disadvantaged and affluent parents are notable and systematic (Bornstein, 2013; Hurt & Betancourt, 2016; Sirin, 2005). Wealthier parents can often better tailor educational activities to their child’s developmental stage (Guryan et al., 2008; Hill & Stafford, 1974; Kalil et al., 2012). They often have more time to engage in reading- and math-related activities and provide diverse, stimulating language interactions that help build vocabulary (Bradley & Corwyn, 2005; Hoff, 2003; Romeo et al., 2013). Low-income parents, despite facing challenges, engage in enriching activities with their children by finding cost-effective and stimulating ways to support development (Cooper, 2021). This is why it is crucial to study the impact of parenting interventions across the socioeconomic gradient, using various measures.

In the United Kingdom, eligibility for FSMs is commonly used as a metric of disadvantage. Students are FSM eligible if their parents receive certain benefits such as unemployment benefit or income support (Department for Education, 2024a). Measures of parental education and occupation level are thought to be the best measures of disadvantage, but they are not widely measured for all students, and FSM eligibility is only marginally less predictive, so is considered one of the better indicators available to researchers (Ilie et al., 2017). However, as FSM is income based, it does not account for all forms of disadvantage. Furthermore, it is a binary variable, not capturing substantial variability within the two groups; students at and around the threshold will not be formally classified as disadvantaged but may well face challenges.

The Income Deprivation Affecting Children Index (IDACI) is a neighborhood-based measure of disadvantage. It is similar on some metrics to the “Child Opportunity Index” in the United States (*Data for a Diverse and Equitable Future |*, n.d.); however to our knowledge, the two have never been directly compared. IDACI reports the proportion of children living in households receiving both in-work and out-of-work benefits relative to the total number of children in that area (Department for Levelling Up, Housing and Communities, 2015). Recent evidence suggests that children in poor neighborhoods receive less rich language exchanges and fewer learning opportunities from adults both at home and at school. This home–school combination constitutes a “double dose of disadvantage” for children during their kindergarten years (Neuman et al., 2018). Finegood et al. (2017) also find that neighborhood disadvantage predicts cortisol levels in a population-based sample of infants and toddlers growing up in low income and rural communities in the United States (Finegood et al., 2017). IDACI has been used previously in conjunction with FSM eligibility to provide more fine-grained analyses across the SES gradient (Clery et al., 2022). This nuanced perspective is vital for understanding whether providing low-cost, easy-to-implement advice can effectively support all parents across the socioeconomic gradient, not just those facing challenges associated with low income (Cooper, 2021).

### EAL

In the United Kingdom, over one-fifth (21.2%) of primary school students speak EAL (Department for Education, 2020). Although this is a sizable proportion of the student population, best practice for how to support non-English-speaking parents varies from school to school (Manzoni & Rolfe, 2019). During the preschool years, children with EAL tend to perform less well on English Language tests (Strand & Lindorff, 2020). This pattern continues into primary school where a consistent achievement gap in language and literacy assessments between EAL children and their monolingual, English-speaking peers is evident (Strand et al., 2015). With ongoing exposure to English, however, the majority of EAL students catch up by the time they reach adolescence (Law et al., 2017).

Examining the impact of Tips by Text on young students with EAL is crucial for several reasons. First, since the text messages were delivered to parents in English, their engagement likely depended on their English proficiency or willingness to use tools like “Google Translate.” This language barrier may have reduced the program’s effectiveness for non-English-speaking parents, resulting in a smaller average treatment effect for EAL students. Second, the assessments were conducted in English, which might have negatively affected EAL students’ performance because of the language challenge rather than developmental delays. We expect overall improvement as students adapt to an English-medium environment, with potentially greater gains in the treatment group if Tips by Text enhanced parental engagement. Finally, Tips by Text may differentially benefit EAL families who might lack access to other resources.

### SEN

In the United Kingdom, the number of students who require support because of SEN has increased by 14% since 2016. Typically, the percentage of SEN students increases with age, peaking at about 20% by age 10 and then declining to around 16% by age 15 (Department for Education, 2024c). Only the most severe cases will have been diagnosed by age 4–5 years (reception year). While it is not possible to compare students who will eventually require SEN support to those who will not, in this study, we can compare students with the most severe SEN cases to all other students.

The parents of SEN students often require intensive, individualized support, which a text message program like Tips by Text may not provide. Despite available online support and tailored information, parents of SEN children report using the internet less frequently and face more barriers than other parents, with 21% finding it too time-consuming compared to 11% of non-SEN parents (Zhang & Livingstone, 2019). There is little consensus on how best to support these families (Gruebner et al., 2022). Exploring the feasibility of an intervention like Tips by Text for parents of SEN children could inform how to effectively share information with this group. While providing simple, friendly activities might be beneficial, it could also be stressful if the activities are not tailored to the child’s needs.

### Multiple Challenges

For a proportion of children, challenges co-occur—for example, a student from a low-income home who does not speak English as their first language and who also has SEN. These students likely constitute a particularly high-risk group, for whom well targeted and more intensive interventions are most likely to have the greatest impact. Defining “disadvantage” is complex and cannot be summatively identified by a select number of variables like FMS, EAL, or SEN (Darbyshire et al., 2014). It may be the result of one factor, or the interaction of several, relating to socioeconomic circumstance, physical and emotional well-being, or prior life events (Krakouer et al., 2017). Albeit not a comprehensive examination of all challenges, this set of analyses contributes to the literature by exploring in depth, the impact of a parent-focused intervention on child early literacy and socioemotional outcomes, and how it may differ in accordance with the type of challenge children experience.

### Study Aims

This study aims to rigorously evaluate the Tips by Text program through a large-scale RCT involving 109 schools and approximately 3,649 reception students (ages 4–5 years). Our goal is to build robust evidence on the effectiveness of regularly sending parents short, actionable tips via short message service that can be easily integrated into daily routines.

We aim to replicate the primary impact analysis conducted by the designated evaluation team (Education Endowment Foundation, 2022) and extend it to investigate how the intervention differentially impacts students facing various challenges, including socioeconomic disadvantage, language barriers, and SEN. Additionally, we explore the effects of the program on students experiencing multiple challenges simultaneously.

This study contributes to the literature on home learning environment interventions by testing a simple, cost-effective method to support parents in a nonintrusive and time-efficient manner. By doing so, it aims to demonstrate how simple, well-timed prompts can enhance parental engagement and improve early childhood development outcomes. We outline our specific hypotheses below.

#### Analysis of Impact: Intervention Effects

*Hypothesis 1:* Students whose parents receive Tips by Text will perform better on early reading (a) and socioemotional well-being (b) measures than the control group.

*Hypothesis 2:* The subgroup of students with below-median baseline scores will perform better on early reading (as observed in York et al., 2019) (a) and socioemotional well-being (b) measures than similar students in the control group.

#### Analysis of Differential Effects by SES

*Hypothesis 3:* The subgroup of FSM-eligible students whose parents receive Tips by Text will perform better on early reading (a) and socioemotional well-being (b) measures than FSM-eligible parents in the control group.

*Hypothesis 4:* The subgroup of students whose parents received Tips by Text and who have higher levels of neighborhood disadvantage will perform better on early reading (a) and socioemotional well-being (b) measures than similar students in the control group.

#### Analysis of Differential Effects by Other Challenges

*Hypothesis 5:* The subgroups of EAL students in the treatment arm will perform differently on early reading (a) and socioemotional well-being (b) measures than similar students in the control group. Note that the direction of the difference is not clear in this case.

*Hypothesis 6:* The subgroups of SEN students in the treatment arm will perform differently on early reading (a) and socioemotional well-being (b) measures than similar students in the control group. Note that the direction of the difference is not clear in this case.

*Hypothesis 7:* Students in multiple subgroups (across FSM, IDACI, EAL, and SEN) in the treatment arm will perform differently on early reading (a) and socioemotional well-being (b) measures than similar students in the control group. Note, again, that the direction of the difference is not clear for these cases.

## Method

### Ethics and Registration

We received ethical approval from the Medical Sciences Interdivisional Research Ethics Committee at the University of Oxford in October 2021 (R77416/RE001). This application detailed the secondary analyses to be conducted.^3^

#### Research Design

The Tips by Text program was evaluated using a two-arm RCT. In total, 109 schools in the Northeast of England were recruited to the RCT from January to July 2019. Preintervention assessments with reception students occurred in September and October 2019. Randomization took place in late October 2019, and the text messages were sent from November 2019 to November 2020. Postintervention testing was supposed to take place from November 2020 to the end of January 2021. About one-third of schools had completed testing by Christmas 2020 with the rest scheduled for January 2021. However, the Department for Education in England closed all schools in early January to curb the spread COVID-19. These closures meant that postintervention data for the primary outcome measure could only be collected for 30% approximately of the sample (*n* = 753). Data collection for the secondary outcome measure was switched to online collection and was therefore less impacted (*n* = 1,037).

#### Sample

Table 1 reports the sample sizes at three different points; the target sample sizes set before school recruitment commenced, the sample size at randomization, and the analytical sample size (postschool closures because of COVID-19). Table 1 also reports the size of subgroups of interest for our planned secondary analysis: children eligible for FSM, children with EAL, and pupils with SEN.[Table tbl1]

Parents and not students were randomized to ensure that families with twins were not allocated to both trial arms. Within each class, half of students’ parents were randomly allocated to receive the text message program (treatment), while the other half did not (“business as usual” control). We acknowledge that without an active control arm, it is difficult to determine whether observed effects are because of the intervention itself or simply increased engagement from receiving text messages. Although we considered sending messages to the control group during the planning phase of the RCT, developing sufficient neutral content for a 12-month program within the available time frame was not feasible. Ideally, a three-arm RCT, comparing the intervention with both passive and active control groups, would better isolate the impact of the content from the effects of engagement through text messaging.

The appointed evaluators carried out the randomization process. Each parent was assigned a random number, and parents within each school were sorted according to this number. The first parent in each sorted list was allocated to either the treatment or control group, with each subsequent parent being allocated to the opposite group of the previous parent. This method ensured an even distribution between the groups and minimized potential biases. Researchers were blinded to the trial arm allocation. We assume that teachers were also blinded to the trial arm allocation of each parent; however, we cannot rule out that in a small number of cases, parents approached teachers to speak about the text messages, which would have revealed the family being in the treatment arm.

The intervention was because of start immediately after the October half-term, 2019. Five schools took their half-term break 1 week earlier than the rest. Randomization was therefore completed in two batches, so that these five could commence the intervention when they returned to school after their break. In schools with multiple reception classes, only one class was randomly selected to complete the primary and secondary outcome assessments. This approach was chosen to reduce the burden on schools and minimize costs and testing time. Out of the *n* = 3,658 students randomized, only *n* = 2,647 were in the selected classes for outcome assessments. The primary reason for missing baseline data on the primary outcome was student absence on the day of testing, with *n* = 2,389 students completing the primary outcome assessment. Teachers completed the secondary outcome measures online for *n* = 1,037 students. See SI 3 in the online supplemental materials for the Consolidated Standards of Reporting Trials participant flow diagram.

#### Measures

##### York Assessment of Reading Comprehension (YARC)

The primary outcome measure was the YARC (Snowling et al., 2009), chosen because it is widely used in the United Kingdom as a tool to assess emerging literacy skills and is similar to the PALS which was used in a previous RCT testing the program in the United States (York et al., 2019). The YARC covers four dimensions of early reading-related skills described in more detail below.

• The sound isolation test measures phoneme isolation skills, which are a component of phonemic awareness. Children heard a series of 12 nonsense words and were asked to identify either the first or the final sound in the word. The test’s internal reliability using Cronbach’s α is .88, and correlation with the single word reading test (SWRT) is .62.
• The sound deletion test measures phoneme deletion skills, which are also a component of phonemic awareness. Children heard a series of 12 words accompanied by a picture of what they represent and were asked to repeat the word but “take away” a sound from the word. The test’s internal reliability using Cronbach’s α is .93, and correlation with the SWRT is .76. If the sound isolation and sound deletion scores are combined, this combined score has an internal reliability of .95 using Cronbach’s α.
• The letter sound knowledge test measures alphabetic knowledge. Children were shown lower case letters and digraphs, one at a time, and were asked to say what sound the letters and digraphs make. The core test comprises 11 letters and six digraphs. The extended test (used in the RCT) comprises 26 letters and six digraphs. The core test’s internal reliability using Cronbach’s α is .95. Its correlation with the SWRT is .55.
• The early word recognition test measures reading attainment in young readers. Children were asked to read 30 single words, which were graded in difficulty. Half of the words had regular correspondence between the graphemes and phonemes, namely, letter to sound mapping, and half were less regular because of inclusion of less common and/or idiosyncratic grapheme–phoneme correspondences and orthographic patterns. The test’s internal reliability using Cronbach’s α is .98, and correlation with the SWRT is .88.

To reduce testing time, students were assessed only on the former two measures at baseline as they are considered the most sensitive for younger children. All four dimensions were used during the postintervention assessment. The YARC was administered by trained researchers who visited schools and conducted one-to-one assessments with each student. A total YARC score that sums the subscales will be standardized (*M* = 0, *SD* = 1). As the scales vary in length, the subscales will then be added together and restandardized.

##### Strengths and Difficulties Questionnaire (SDQ)

The secondary outcome measure was the SDQ, a widely used behavioral screening tool for assessing behaviors, emotions, and relationships in children aged 2–18 (R. Goodman, 2001). Originally, the SDQ was developed to assess strengths and difficulties in children with potential mental health concerns, such as emotional symptoms. However, it has increasingly been used to capture variations in well-being within the general population. The teacher version has shown strong psychometric properties with Cronbach’s α ranging from .70 to .80 and test–retest reliability correlations above .70 (Stone et al., 2010).

The SDQ includes 25 items across five scales: emotional symptoms, conduct problems, hyperactivity/inattention, peer relationship problems, and prosocial behavior. We used the “total difficulties” score, which comprises all scales except prosocial behavior, resulting in a score ranging from 0 to 40. We also conducted separate analyses for three subcategories: “internalizing problems” (comprising the emotional symptoms and peer relationship problems scales), “externalizing problems” (comprising the conduct problems and hyperactivity scales), and “prosociality” (the prosocial scale; R. Goodman, 2001). Following standard SDQ conventions (Vaz et al., 2016), if at least three of the five items on a subscale are completed, missing scores will be replaced by the subscale mean. If fewer than three items are completed, no score will be calculated for that subscale, and if any of the four subscales are missing, the total difficulties score will not be computed.

The primary and secondary outcome measures were selected in collaboration with the funder and the appointed evaluators. Due to cost implications and the feasibility of implementing multiple measures across 109 schools within a relatively short testing window, only two measures could be implemented. Because the intervention targets numeracy as well as language/literacy and socioemotional skills, we planned to supplement the primary and secondary measures with administrative data collected by the Department for Education in the United Kingdom, specifically, the literacy, numeracy, and social development subscales of the EYFSP (Department for Education, 2023). However, the EYFSP was cancelled in 2020 and 2021 because of the COVID-19 pandemic, preventing us from conducting these analyses.

##### FSMs

Students are eligible for FSMs if their parents are in receipt of certain benefits (e.g., income support; Department for Education, 2024a). FSM is a binary variable, with 1 denoting *eligibility* and 0 denoting *otherwise*.

##### IDACI

IDACI is a U.K.-based metric that ranks lower super output areas by the percentage of children aged 0–15 living in income-deprived families. Lower super output areas are defined geographic units with approximately 1,000 residents each. The IDACI ranking ranges from 1 (*most deprived*) to 32,482 (*least deprived*; Department for Levelling Up, Housing and Communities, 2015).

##### EAL

The text messages were developed using the most simple and accessible language possible to ensure parents whose first language is not English could still engage with the intervention. Inevitably, there were some who still could not discern the language, and so this analysis is of interest to further our understanding of which intervention types are useful for this subset of parents. EAL is a binary variable; 1 denoting if *English is spoken as a second language in the household* and 0 if *otherwise*.

##### SEN

We expect a small number of SEN to be present in the sample. During the school recruitment phase, it was emphasized to all schools that SEN students were not required to either partake in the baseline and end line assessments nor were their parents required to receive the text messages if they did not want to. Like EAL and FSM, SEN is also a binary variable; 1 if the *child has SEN* and 0 if *they do not*.

##### Multiple Challenges

We will create a subgroup of children experiencing multiple challenges, that is, they are eligible for FSM, are living in an area ranking high on IDACI, speak EAL, and have SEN. We will use a median split on IDACI scores to divide the sample into two groups, thus creating a binary variable. We will then create a cumulative challenge score for each student by adding up the respective scores on each measure.

#### Intervention

Tips by Text is a 12-month text messaging intervention for parents of Reception students (aged 4–5 years). The activities target language, literacy, socioemotional, and numeracy skills. The split of content was 50% language and early literacy, 25% socioemotional skill development, and 25% numeracy. Parents received the “FACT” and “TIP” messages at 3.45 p.m. on Mondays and Wednesdays, respectively, and the “GROWTH” message at 10.30 a.m. on Saturdays.

#### Piloting

We adapted the program for use in the United Kingdom from its original version, which was first tested in the United States. To do this, we conducted two rounds of piloting with teachers and parents. Participants were recruited from schools that were not partaking in the trial. The pilots were designed to gather information around three themes—(a) content: ensuring the activities were accessible and pitched at the right level for UK-based reception students; (b) context: understanding existing home routines and parental behaviors to ensure the intervention would be compatible; and (c) delivery: sense-checking practical aspects of delivery during the trial such as the time of day to send the messages and the potential for message sharing to occur.

Pilot 1 was conducted from March to May 2019. One school that had volunteered for the pilot circulated a notice to parents with the relevant details. Ten parents consented to receiving text messages for 2 weeks (six messages in total) and *n* = 8 parents completed a follow-up interview. Teachers were recruited from a small pool of schools who were interested in partaking in the trial but did not meet the criteria. Ten teachers were sent a selection of text messages via an online survey (3 weeks’ worth of content; nine messages in total) and were asked to comment in line with an accompanying set of instructions. Eight teachers could be reached for the follow-up interview.

The same process was repeated during a second round of piloting (June to July 2019) with a larger group of parents (survey responses: *n* = 19 and completed interviews: *n* = 12) and teachers (survey responses: *n* = 19 and completed interviews: *n* = 12). The findings are summarized below.

• Content: Parents felt the language, phrasing and tone of the messages were appropriate, and signaled that they thought parents would find the content useful and accessible. Parents also appreciated that the activities did not require additional resources. Some parents mentioned the activities were potentially too easy for their children. We interpreted this feedback with a degree of caution given the potential for selection bias in the pilot (i.e., parents who volunteered were likely already very engaged in their child’s education). However, it does prompt consideration for future research to explore the possibility of running differentiated programs for lower and higher achievers. Teachers reported the program being well aligned to the early years foundation stage framework, as well as the activities being complementary to what they were teaching in class.
• Context: Parents thought that the Tips by Text activities would fit well in their daily routines and were complementary to the types of activities they do with their children already, like going to the park or doing arts and crafts at home. Parents described their daily routines as hectic, with mornings focused on preparing for school and evenings involving homework, playtime, and bedtime rituals. Parents valued learning, education, and emotional connections for their children’s development, often supplementing school efforts through activities such as shared book reading. Teachers believed that most parents would engage with the Tips by Text program but had mixed views on whether text messages would lead to improvements on an objective measure like early reading skills.
• Delivery: Parents were asked about their preferred times for receiving messages, with responses varying from early morning to late evening. Many preferred receiving texts after school, so messages were scheduled for 3:45 p.m. on Tuesdays and Thursdays, and 10:30 a.m. on Saturdays. Teachers supported these timings. Regarding message sharing, parents had mixed views: some believed sharing may occur among friends, while others said they had not considered sharing the messages until it was mentioned by the interviewer.

After each round of piloting, the text messages were updated to reflect feedback from parents and teachers. A final review of the program occurred in September 2019 ahead of the trial launching in November.

#### Texting Platform

Following the consent procedure, schools shared the telephone numbers of parents, which were inputted into the texting platform. If text messages failed to deliver after nine consecutive attempts, a flag would be raised. We then followed up with the relevant school, which contacted the parent to obtain a new telephone number. This new number was then uploaded to the platform and parents would recommence receiving messages for that week (i.e., we did not attempt to resend the messages they had missed out on). All parents enrolled in the trial could opt out at any point by texting “STOP” in response to any text message. Parents could respond to the text messages if they wished. However, they would not receive a response from the research team in return. This lack of response was to avoid researchers getting into a two-way conversation with parents. A member of the research team checked the platform every morning for any responses that could raise a safeguarding concern. In such cases, we contacted the relevant school immediately.

### Planned Statistical Analysis

Our planned analyses were divided into three sets. The first set examined the overall impact of the intervention on primary and secondary outcomes, as well as differences between high and low attainers. The appointed evaluation team reported no significant findings for this set of analyses (Education Endowment Foundation, 2022); however, we include them here for completeness. The second set of analyses examined the varying effects based on socioeconomic challenge, using FSM eligibility (also investigated by the independent evaluators; Education Endowment Foundation, 2022) and further extending this analysis by including IDACI scores. The third set investigated the intervention’s effects on students with EAL and SEN, as well as those facing multiple challenges. The second and third sets were novel and aimed to enhance the impact analysis by providing a more in-depth understanding of how the Tips by Text program differentially affects students experiencing various forms of challenge.

Impact was estimated using an intention-to-treat analysis regardless of dropout. We ran multilevel mixed-effects models to account for the hierarchical structure of the data, where students were nested within schools. These models treated the effect of the school as a random variable to appropriately capture the variability between schools. All models were run in R software Version 4.3.1 (R Core Team, 2024) using the package “lmer.” We conducted a false discovery rate (FDR) correction to adjust for multiple comparisons and control the rate of Type I errors. We included age (in months) and gender in all models to control for natural developmental differences and gender-related variation; full data for these two variables were available.

For the subgroups (FSM, EAL, SEN, and multiple challenges), we created separate groups (e.g., pupils receiving FSM vs. those who were not). This allowed us to examine how the intervention effects varied across predefined groups within the study population. We also conducted moderation analysis to investigate whether the relationship between the treatment and the primary and secondary outcomes was influenced by a third variable.

Although parents who participated in the pilot phase indicated they would not share messages with other parents, it remains theoretically possible that parents in the control group could have received text messages because of parental sharing. To address this, we conducted a sensitivity analysis to assess the robustness of our results to potential spillover effects by systematically varying assumptions about the degree of spillover (i.e., creating high and low spillover groups) and reanalyzing the data under each scenario. Regarding dosage, during the 12-month intervention period, 165 messages were sent to parents in the treatment group and 81% received at least 150 of these messages, indicating a high level of fidelity. There is therefore limited scope for further analysis based on dosage as variation across the sample is minimal.

#### Data Sources

Data collected directly by the appointed evaluators during the RCT were shared for the purpose of conducting secondary data analysis. We retrospectively obtained additional variables from the National Pupil Database (NPD), a register data set of all pupils in state schools in England, managed by the Department for Education (Department for Education, 2024b). The NPD contains attainment data as children progress through school, as well as information on student background, absences, and exclusions from school. We will obtain the following variables from the NPD: FSM, IDACI, EAL, and SEN to conduct additional subgroup and moderation analysis (Table 2).[Table tbl2]

#### Attrition

The nationwide school closures in England in early 2021 because of COVID-19 led to a significant reduction in the analysis sample size. As this attrition resulted from an exogenous event that affected all schools equally, it was assumed to be random. However, to validate this assumption, we examined whether missing data were systematically associated with specific school and pupil characteristics. We conducted logistic regression analyses using a binary indicator of missingness as the outcome, with predictors including school effectiveness (e.g., Ofsted rating), school location, type, and pupil demographics (percentage of pupils eligible for FSM in the past 6 years, percentage of pupils with EAL, and percentage of pupils with SEN). Additionally, we checked that missingness was balanced across trial arms to ensure that any bias in attrition did not differentially affect the treatment and control groups. Despite this reduction, the remaining sample size was substantial (*n* = 753) and comparable to the efficacy study by York et al. (2019). Of the 2,647 students eligible for testing, 2,389 completed the YARC at baseline, and 781 completed it postintervention. The sample size for the SDQ was *n* = 1,037. Refer to SI 3 in the online supplemental materials for the Consolidated Standards of Reporting Trials participant flow diagram.

We considered using full information maximum likelihood for handling missing data. However, the baseline data set was limited to standard demographic variables and the YARC, providing insufficient auxiliary variables for effective estimation. Additionally, the high attrition rate of approximately 70% rendered full information maximum likelihood unsuitable for our sample. We decided to proceed with listwise deletion of participants who did not have both fully completed baseline and end line YARC assessments.

#### Analysis of Impact: Intervention Effects

We report descriptive statistics including sample sizes, means, and standard deviations for the following continuous variables: age, IDACI, and baseline YARC scores. For binary variables (FSM eligibility, students with EAL, and students with SEN), frequencies are reported for each category (e.g., eligible vs. non-eligible for FSM, students with EAL vs. without, students with SEN vs. without). For comparison, we include statistics for three groups: the sample at randomization (the randomized sample), the sample selected to complete baseline assessments (the assessed sample), and the sample with both baseline and end line data (the analysis sample). Additionally, YARC scores at baseline and end line are reported for each subgroup, including means, standard deviations, and other relevant statistics within each group. We also provide school level demographics including Ofsted ratings (an education quality metric used in the United Kingdom), school type, urban/rural location, percentage FSM students, percentage EAL students, percentage SEN students, and the average school level performance at the end of Key Stage 2 (age 11).

##### Primary Outcome: The YARC (Hypothesis 1a)

We estimated the following equation to understand the impact of the treatment on the YARC:

$$\begin{array}{rl} Y_{ijt}= & \alpha +\beta_{1}{\mathrm{Treat}}_{i}+\beta_{2}T_{ijt-1}+\beta_{3}y_{j}+\beta_{4}{\mathrm{age}}_{i}+\beta_{5}{\mathrm{gender}}_{i}\\ & +u_{j}+\varepsilon_{ijt}, \end{array}$$ 1

where *i* are students and *j* are schools, *Y_ijt_* is the YARC posttest score, α is the overall intercept, β_1_Treat*_i_* is the fixed effect of the treatment indicator for student *i* (1 represents being *allocated to the treatment group* and 0 represents *allocation to the control group*), β_2_*Y_ijt_*_−1_ is the fixed effect of the YARC pretest score for student *i*, β_3_ *y_j_* is the fixed effect of the randomization batch for school *j*, β_4_age is the fixed effect of the age in months at baseline for student *i*, β_5_gender is the fixed effect of the gender of student *i*, *u_j_* is the random intercept for school *j*, and ε*_ijt_* is the residual error term. Estimated impact (Treat*_i_*) was converted into a Hedges’ *g* effect size. This measure used the estimated total pooled standard deviation of the treatment and control groups rather than the within-school pooled standard deviation as the former approach is more conservative.

##### Secondary Outcome: SDQ (Hypothesis 1b)

The “total difficulties” score was constructed by summing together responses from four of the five SDQ subscales—emotional problems, conduct problems, hyperactivity, and peer problems. Separate models were run for the three subcategories “internalizing problems,” “externalizing problems,” and the prosocial scale (A. Goodman et al., 2010). The analysis of the secondary outcome followed the same approach used for the primary outcome. As determined by the independent evaluation team, the SDQ was not collected at baseline. We therefore used baseline YARC scores as a control variable to account for differences between pupils in the two trial arms. Although the absence of baseline SDQ data were not ideal, using YARC scores was preferable to not controlling for potential baseline differences and relying solely on randomization to account for them. We anticipated a correlation between individual differences on the YARC and the SDQ. As a sensitivity check, we also ran models with and without baseline YARC scores and compared the resulting estimates. The estimated equation was:

$$\begin{array}{rl} Ysdq_{ijt}= & \alpha +\beta_{1}{\mathrm{Treat}}_{i}+\beta_{2}Y_{ijt-1}+\beta_{3}y_{j}+\beta_{4}{\mathrm{age}}_{i}\\ & +\beta_{5}{\mathrm{gender}}_{i}+u_{j}+\varepsilon_{ijt}, \end{array}$$ 2

where *Ysdq_ijt_* is the total difficulties score and all other elements were as defined above. Estimated impact was converted into a Hedges’ *g* effect size as before.

##### High Versus Low Prior Attainers (Hypothesis 2a)

We assessed whether the intervention differentially affected students with high versus low baseline attainment, using YARC scores. Students were divided into high and low attainers based on their median YARC score at baseline, following the method used by York et al. (2019). We fitted a mixed-effects model with an interaction term between treatment and attainment, incorporating the high and low attainers variable. We then computed summary statistics for both the treatment and control groups within each attainer subgroup. Additionally, we calculated Hedges’ *g* and its confidence intervals (CIs) for both the high attainers and low attainers subgroups. The estimated equation for the primary outcome (hypothesis 2a) was:

$$\begin{array}{rl} Y_{ijt}= & \alpha +\beta_{1}{\mathrm{Treat}}_{i}+\beta_{2}Y_{ijt-1}+\beta_{3}y_{j}+\beta_{4}{\mathrm{Att}}_{i}+\beta_{5}{\mathrm{Treat}}_{i}\\ & \times {\mathrm{Att}}_{i}+\beta_{6}{\mathrm{age}}_{i}+\beta_{7}{\mathrm{gender}}_{i}+u_{j}+\varepsilon_{ijt}, \end{array}$$ 3

where β_4_Att*_i_* is the effect of the attainment level (high/low) and β_5_Treat*_i_* × Att*_i_* is the interaction effect between the treatment and attainment. All other elements were as defined above.

The same model was run for the secondary outcome measure—socioemotional well-being (hypothesis 2b). We focused on whether the Treatment × Attainment interaction was significant because that would indicate whether the treatment had a differential effect for students with varying levels of attainment. When an interaction was found, we generated plots to visualize the outcome by attainment group.

#### Analysis of Differential Effects by SES

##### FSMs

We conducted a subgroup analysis of FSM-eligible students for both the primary and secondary outcomes. The data were filtered to create two subgroups: those eligible for FSM and those not eligible. Separate mixed-effects models were run for each subgroup. Additionally, we ran a model with an interaction term between treatment and FSM eligibility to assess whether the intervention had a differential effect based on FSM status. Running these models provided a comprehensive understanding of the treatment effects within each subgroup and determined if the intervention’s impact varied between different socioeconomic groups. The focus was on whether the Treatment × FSM interaction effect was significant, as this would indicate a differing treatment effect for the FSM-eligible group. If the interaction effect was statistically significant, we generated plots to examine the outcomes by the FSM group. The estimated equation was identified as primary outcome: early reading (Hypothesis 3a):

$$\begin{array}{rl} Y_{ijt}= & \alpha +\beta_{1}{\mathrm{Treat}}_{i}+\beta_{2}Y_{ijt-1}+\beta_{3}y_{j}+\beta_{4}{\mathrm{FSM}}_{i}+\beta_{5}{\mathrm{Treat}}_{i}\\ & \times {\mathrm{FSM}}_{i}+\beta_{6}{\mathrm{age}}_{i}+\beta_{7}{\mathrm{gender}}_{i}+u_{j}+\varepsilon_{ijt}, \end{array}$$ 4

where β_4_FSM*_i_* is the effect of FSM and β_5_Treat × FSM*_i_* is the interaction effect between the treatment and FSM. All other elements were as defined above. We ran the same model for the secondary outcome measure—socioemotional well-being (Hypothesis 3b).

##### IDACI

IDACI is a continuous variable measuring neighborhood disadvantage. We ran a model interacting the treatment with IDACI. We were interested in the coefficient of the interaction effect, which estimates the relationship between the treatment effect and area-based disadvantage. The estimated equation was identified as primary outcome: YARC (Hypothesis 4a):

$$\begin{array}{rl} Y_{ijt}= & \alpha +\beta_{1}{\mathrm{Treat}}_{i}+\beta_{2}Y_{ijt-1}+\beta_{3}y_{j}+\beta_{4}{\mathrm{IDACI}}_{i}\\ & +\beta_{5}\;{\mathrm{Treat}}_{i}\times {\mathrm{IDACI}}_{i}+\beta_{6}{\mathrm{age}}_{i}+\beta_{7}{\mathrm{gender}}_{i}+u_{j}+\varepsilon_{ijt}, \end{array}$$ 5

where β_4_IDACI*_i_* is the effect of IDACI and β_5_Treat × IDACI*_i_* is the interaction effect between the treatment and IDACI. All other elements were as defined above. We ran the same model for the secondary outcome measure—socioemotional well-being (Hypothesis 4b).

#### Analysis of Differential Effects by Other Challenges

We examined whether the intervention had a differential impact on students with EAL and SEN and if multiple challenges cumulatively moderated the intervention’s effect.

##### EAL

We created two subgroups: EAL and non-EAL and ran separate models for each to obtain summary statistics for the treatment and control groups. We also ran a model with an interaction term between the treatment and EAL. The estimated equation was identified as primary outcome: YARC (Hypothesis 5a):

$$\begin{array}{rl} Y_{ijt}= & \alpha +\beta_{1}{\mathrm{Treat}}_{i}+\beta_{2}Y_{ijt-1}+\beta_{3}y_{j}+\beta_{4}{\mathrm{EAL}}_{i}+\beta_{5}{\mathrm{Treat}}_{i}\\ & \times {\mathrm{EAL}}_{i}+\beta_{6}{\mathrm{age}}_{i}+\beta_{7}{\mathrm{gender}}_{i}+u_{j}+\varepsilon_{ijt}, \end{array}$$ 6

where β_4_EAL*_i_* is the effect of EAL and β_5_Treat × EAL*_i_* is the interaction effect between the treatment and EAL. All other elements were as defined above. We ran the same model for the secondary outcome measure—socioemotional well-being (Hypothesis 5b).

##### SEN

As with EAL, data were filtered to create two subgroups: SEN and non-SEN. We ran separate models for each group. We also ran a model interacting the treatment allocation with SEN. The estimated equation was identified as primary outcome: YARC (Hypothesis 6a):

$$\begin{array}{rl} YS_{ijt}= & \alpha +\beta_{1}{\mathrm{Treat}}_{i}+\beta_{2}Y_{ijt-1}+\beta_{3}y_{j}+\beta_{4}{\mathrm{SEN}}_{i}+\beta_{5}{\mathrm{Treat}}_{i}\\ & \times {\mathrm{SEN}}_{i}+\beta_{6}{\mathrm{age}}_{i}+\beta_{7}{\mathrm{gender}}_{i}+u_{j}+\varepsilon_{ijt}. \end{array}$$ 7

We ran the same model for the secondary outcome measure—socioemotional well-being (Hypothesis 6b).

##### Multiple Challenges

For each challenge measure (FSM, IDACI, EAL, and SEN), we created a binary variable and then summed them to generate a cumulative score for each student. We specified a median cutoff for the continuous variable IDACI. Contingent on an adequate sample size, we planned to investigate the interaction between the treatment and the multiple challenges score. The planned equation to be estimated was identified as primary outcome: YARC (Hypothesis 7a):

$$\begin{array}{rl} Y_{ijt}= & \alpha +\beta_{1}{\mathrm{Treat}}_{i}+\beta_{2}Y_{ijt-1}+\beta_{3}y_{j}+\beta_{4}{\mathrm{MulVul}}_{i}+\beta_{5}{\mathrm{Treat}}_{i}\\ & \times {\mathrm{MulVul}}_{i}+\beta_{6}{\mathrm{age}}_{i}+\beta_{7}{\mathrm{gender}}_{i}+u_{j}+\varepsilon_{ijt}. \end{array}$$ 8

We planned to estimate the same equation for the secondary outcome measure—socioemotional well-being (Hypothesis 7b).

## Results

To assess baseline equivalence between the intervention and control groups, we first examined descriptive statistics for key demographic and outcome variables prior to the intervention, presented in Table 3. The randomized sample consisted of 3,649 participants, with 2,389 completing the YARC assessment at baseline. The intervention (*n* = 1,185) and control (*n* = 1,204) groups were well balanced across key demographic variables, including IDACI scores, age, gender, FSM eligibility, EAL status, and SEN status. Mean IDACI scores, representing neighborhood disadvantage, were similar between groups, as were the distributions of age and gender. The rates of FSM eligibility, EAL, and SEN were also comparable, suggesting that randomization was successful in achieving baseline equivalence between groups. Refer to SI 5 in the online supplemental materials for a detailed breakdown of key variables across trial arms. These findings indicate that any differences in outcomes can reasonably be attributed to the intervention rather than preexisting group differences.[Table tbl3]

To examine whether missingness in postintervention assessments was systematically related to trial arm assignment, we conducted a logistic regression analysis using a binary indicator of missingness (0 = *missing posttest score*, 1 = *completed posttest*) as the outcome. Predictors included school-level characteristics (Ofsted rating and school location) and pupil demographics (percentage of FSM eligible students in the past 6 years, percentage of students with EAL, and percentage of students with SEN). Additionally, we tested for differential attrition by trial arm to assess the risk of bias because of selective dropout. A Pearson’s chi-square test with Yates’ continuity correction indicated that missingness did not significantly differ between the treatment and control groups, χ^2^(1) = 0.008, *p* = .929. This suggests that the likelihood of missing data were balanced across conditions, reducing concerns about systematic attrition bias. Additional details on the school sample composition and missing data patterns can be found in SI 6 in the online supplemental materials.

### Analysis of Impact: Intervention Effects

Our impact analysis assessed whether students whose parents received the text messages showed improvements on the primary outcome, the YARC (Hypothesis 1a), and the secondary outcome, the SDQ (Hypothesis 1b). We compared the average treatment effect between groups and calculated Hedge’s *g* effect size. The results provided no evidence that the intervention influenced either outcome. Table 4 presents the unadjusted means, standard deviations, and Hedge’s *g* effect size for the primary and secondary outcomes.[Table tbl4]

Next, we examined whether students with lower baseline YARC scores performed better on the primary and secondary outcomes, as was reported in the original study (York et al., 2019). Splitting the sample at the median baseline score, we did not find evidence that students scoring below the median showed greater improvements on the YARC (Hypothesis 2a) or on the SDQ total difficulties (Hypothesis 2b; see Table 5). Additionally, we tested models that included an interaction term (Treatment × Low Achievers) for both the YARC and SDQ but found no evidence of an effect (see Table 6).[Table tbl5][Table tbl6]

### Analysis of Differential Effects by SES

We then investigated whether intervention effects differed based on socioeconomic disadvantage, using FSM eligibility and IDACI scores as indicators. See Table 7 for the results. For FSM-eligible students, the intervention had no effect on the YARC (Hypothesis 3a; β = 0.097, *p* = .43; Hedges’ *g* = 0.093) nor on SDQ total difficulties scores (Hypothesis 3b; Hedges’ *g* = −0.041, *p* = .707). Similarly, FSM noneligible students showed no significant intervention effects for either outcome.[Table tbl7]

The full-sample interaction model (see Table 8) confirmed that FSM-eligible students had significantly lower YARC scores overall (β = −0.268, *p* = .004), highlighting an SES-related literacy gap. However, the intervention did not differentially affect FSM and non-FSM students (Treatment × FSM interaction: β = 0.058, *p* = .652), indicating no significant moderating effect of FSM eligibility on YARC outcomes. For SDQ total difficulties (*n* = 1,124), we did not find a main effect of the intervention (β = 0.72, *SE* = 0.37, *p* = .52). FSM-eligible students had significantly higher SDQ difficulties overall (β = 1.30, *SE* = 0.50, *p* = .01), reinforcing the link between socioeconomic disadvantage and socioemotional challenges. However, the Treatment × FSM interaction was not statistically significant (β = −0.96, *SE* = 0.69, *p* = .162), suggesting the intervention’s effect on SDQ scores did not differ significantly between FSM-eligible and non-FSM students. Graphs for all interaction models are included in SI 7 in the online supplemental materials.[Table tbl8]

When examining neighborhood disadvantage, we tested whether IDACI scores moderated the intervention’s effect on YARC (Hypothesis 4a) and SDQ total difficulties (Hypothesis 4b) in the full-sample interaction model. See Table 9 for results. No significant main effect of the intervention was found on YARC scores (β = −0.149, *SE* = 0.132, *p* = .256), and the Treatment × IDACI interaction was also not statistically significant (β = 0.621, *SE* = 0.434, *p* = .152), indicating that neighborhood disadvantage did not moderate reading-related outcomes.[Table tbl9]

For SDQ total difficulties, we did not find a main effect of either the treatment or IDACI (see Table 9). However, we found a marginally significant Treatment × IDACI interaction (β = 4.899, *SE* = 2.476, *p* = .048), suggesting that the intervention’s effect on socioemotional difficulties varied by socioeconomic background.

To further investigate the interaction between treatment and IDACI score on SDQ total difficulties, we conducted a simple slopes analysis at representative values of IDACI (0.1, 0.3, 0.5, and 0.7) and a Johnson–Neyman (JN) test to determine the range where the interaction effect was statistically significant. The simple slopes analysis examined treatment effects across different levels of IDACI score. At lower IDACI values (0.1 and 0.3), indicating lower levels of income deprivation among children, the estimated marginal means for the treatment and control groups were similar, with overlapping 95% CIs, suggesting no significant difference between the groups. At higher IDACI values (0.5 and 0.7) indicating high income deprivation, the treatment group exhibited higher SDQ total difficulties scores compared to the control group (see SI 8 in the online supplemental materials for a graphical representation). However, despite this apparent trend, the CIs remained overlapping, meaning the treatment effect was not statistically significant at any of the examined IDACI levels. To explore whether the treatment effect varied across the full range of IDACI scores, we conducted a JN test. The test indicated that the interaction effect would be statistically significant when values for “trial arm” were outside the interval [−22.85, 1.25]. However, given that the observed values of “trial arm” were limited to [0, 1], denoting control and treatment respectively, this suggests that no significant interaction effect was detected within the observed IDACI range.

**Figure 1 fig1:**
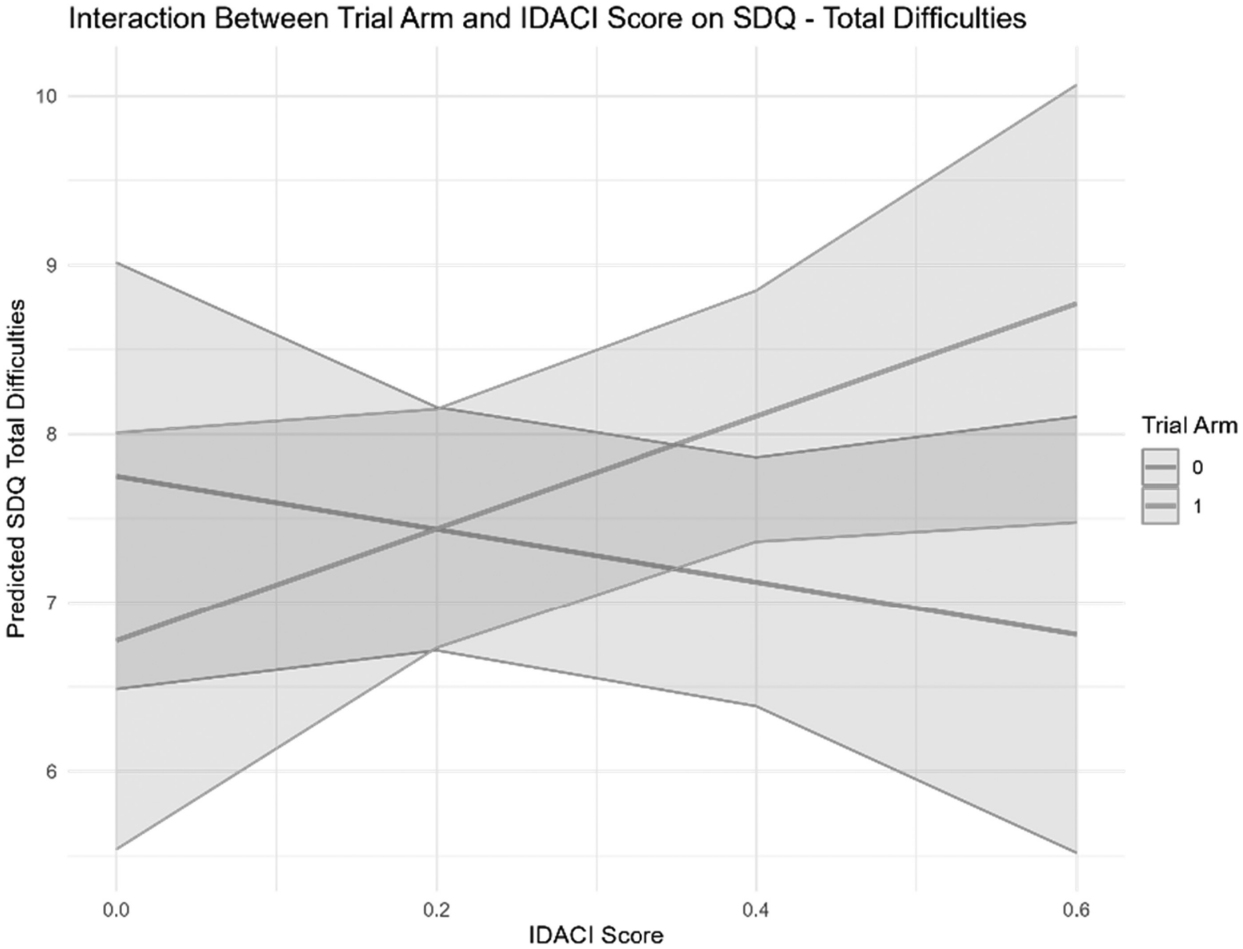
Treatment × IDACI Interaction—SDQ Total Difficulties *Note.* IDACI = Income Deprivation Affecting Children Index; SDQ = Strengths and Difficulties Questionnaire.

Overall, these analyses indicate that the intervention did not produce a statistically significant effect on SDQ total difficulties at any specific IDACI level. Although a trend emerged suggesting that students from more disadvantaged neighborhoods (higher IDACI) may have experienced increased socioemotional difficulties following the intervention (see Figure 1), this pattern did not reach statistical significance. The absence of a significant JN interval further suggests that the observed interaction effect is not robust within the current sample. Given the relatively low number of students in the analysis sample (*n* = 71/1,121) experiencing higher deprivation, as indexed by an IDACI score of greater than 0.5, statistical power to detect an interaction effect may have been limited. Future research should reassess this relationship in a larger sample to determine whether the observed trend is replicable and whether additional contextual factors influence the differential impact of the intervention across socioeconomic backgrounds.[Fig fig1]

### Analysis of Differential Effects by Other Challenges

The third set of analyses focused on the intervention’s effects on students with EAL and SEN, as well as those facing multiple challenges. These analyses built on the second set, offering a deeper investigation of how the Tips by Text program may impact students experiencing different forms of disadvantage.

#### EAL

To assess whether the intervention had differential effects on EAL students, we ran linear mixed-effects models predicting posttest YARC (Hypothesis 5a) and SDQ total difficulties scores (Hypothesis 5b), including a random intercept for school. As with the FSM subgroup analysis, we conducted three models: a subgroup analysis for EAL students, a subgroup analysis for non-EAL students, and a full-sample interaction model testing whether intervention effects differed by EAL status.

For EAL students (*n* = 68), the intervention showed a marginally significant, positive effect on reading-related skills (β = 0.382, *SE* = 0.191, *p* = .045; Hedges’ *g* = 0.375), indicating a trend toward greater gains in this domain among EAL students who received the intervention. However, after adjusting for multiple comparisons, the *p* value was no longer statistically significant at the α = .05 level (see SI 10 in the online supplemental materials for a comparison of *p* values).

We did not find a significant effect of the intervention on reducing socioemotional difficulties (β = −2.013, *SE* = 1.117, *p* = .076; Hedges’ *g* = −0.379). See Table 10 for full results. For non-EAL students (*n* = 685), the intervention had no significant effect on YARC (β = −0.010, *SE* = 0.060, *p* = .865; Hedges’ *g* = −0.010) or on SDQ total difficulties (β = 0.586, *SE* = 0.324, *p* = .071; Hedges’ *g* = 0.106). Across all models, baseline reading-related skills were the strongest predictor of YARC scores (β = 0.505–0.643, *p* < .001) and were also associated with lower SDQ total difficulties. Gender differences were observed, with girls performing better on YARC, and boys showing higher SDQ total difficulties.[Table tbl10]

**Figure 2 fig2:**
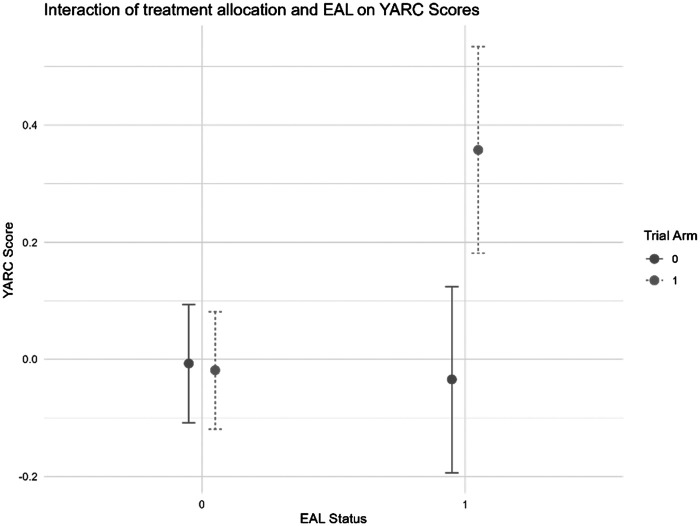
Effect of Treatment × EAL Interaction on YARC *Note.* EAL = English as an additional language; YARC = York Assessment of Reading Comprehension.

To examine whether intervention effects differed by EAL status, we ran full-sample interaction models for both YARC and SDQ (see Table 11 for results). For YARC (*n* = 753), consistent with the findings for the EAL group alone, a significant interaction between the treatment and EAL (β = 0.404, *SE* = 0.203, *p* = .047) suggests that EAL students in the treatment group showed greater improvements than their non-EAL peers. However, as before, after adjusting for multiple comparisons, this effect was no longer statistically significant (Figure 2).[Table tbl11][Fig fig2]

For SDQ total difficulties (*n* = 1,124), the Treatment × EAL interaction was not significant (β = −1.843, *SE* = 1.182, *p* = .119), indicating that the intervention’s impact on SDQ difficulties did not differ between EAL and non-EAL students.

#### SEN

To examine whether the intervention effects differed by SEN status, we conducted the same subgroup analyses as for EAL students, assessing both YARC scores (Hypothesis 6a) and SDQ total difficulties (Hypothesis 6b; see Table 12 for results).[Table tbl12]

For students with SEN, the intervention had no significant effect on YARC scores (Hedges’ *g* = −0.010, 95% CI = [−0.427, 0.408], *p* = .964) or SDQ total difficulties (Hedges’ *g* = −0.137, 95% CI = [−0.492, 0.219], *p* = .452). Similarly, among non-SEN students, the intervention had no significant impact on YARC scores (Hedges’ *g* = 0.037, 95% CI = [−0.084, 0.158], *p* = .552) or SDQ total difficulties (Hedges’ *g* = 0.107, 95% CI = [−0.009, 0.223], *p* = .070).

The interaction models examined whether SEN status moderated the intervention’s effects on YARC and SDQ total difficulties (see Table 13). For YARC, there was no significant Treatment × SEN interaction (β = −0.075, *SE* = 0.196, *p* = .703), indicating that the intervention’s impact on reading-related outcomes did not differ between SEN and non-SEN students. For SDQ total difficulties, the Treatment × SEN interaction was also not statistically significant (β = −1.061, *SE* = 0.940, *p* = .260), suggesting that the intervention’s effect on socioemotional difficulties did not vary by SEN status.[Table tbl13]

Overall, these findings suggest that the intervention did not significantly influence reading or socioemotional outcomes for SEN students. Additionally, there was no evidence of differential effects based on SEN status, as indicated by nonsignificant interaction terms for both YARC and SDQ outcomes.

#### Multiple Challenges

We preregistered analyses to examine the program’s impact on students facing multiple challenges, including those who are economically disadvantaged, have EAL, and have SEN (Hypotheses 7a and 7b). However, after filtering the sample to identify students meeting all these criteria, we found the sample size was too small to yield reliable results. As a result, we did not proceed with this analysis.

### Robustness Checks

#### Estimating Spillover Effects on the Primary Outcome (YARC Scores)

Given the nature of the intervention—text messages sent to parents—it is plausible that spillover may have occurred if parents in the treatment group shared intervention content with those in the control group. To assess the potential impact of such spillover, we estimated how different levels of spillover (i.e., 10% and 20% of the control group receiving indirect exposure to the intervention) might affect the treatment effect size, using Hedges’ *g* as our primary measure. To quantify spillover, we first computed the proportion of treated pupils within each school and used this as a continuous measure of potential exposure among the control group. A mixed-effects model was fit to the full sample to estimate the main treatment effect on YARC scores, controlling for baseline YARC scores, child age, gender, and school-level clustering. To simulate the impact of spillover, we adjusted the control group’s outcome scores by reassigning a proportion (10% and 20%) of control group children as effectively “treated”—reflecting the assumption that they were indirectly exposed to intervention content. The adjusted data set was then analyzed using the same mixed-effects model as the main impact analysis, ensuring consistency in estimation.

In the absence of spillover, the estimated treatment effect size (Hedges’ *g*) was 0.0200. When 10% of the control group was assumed to be indirectly exposed to the intervention, the effect size declined to 0.0136, and with 20% spillover, it further declined to 0.0072. These results suggest that even modest levels of spillover could attenuate the estimated treatment effect, potentially biasing results toward the null. Overall, while the intervention effect on YARC scores remained small, the spillover analysis suggests that diffusion of intervention materials among parents could partially explain the attenuated treatment effects observed in the main analysis. These findings highlight the need for careful consideration of spillover when designing and interpreting trials of parent-targeted interventions.

#### Accounting for Multiple Comparisons

To account for multiple comparisons, we applied an FDR correction, after which none of the subgroup effects remained statistically significant at the α = .05 level. Before correction, there was a trend suggesting that EAL students in the treatment group showed greater improvements on the YARC (*p* = .045). While the FDR-adjusted *p* values exceeded .05, indicating that these findings may be false positives, they may also reflect meaningful patterns that warrant further investigation. Given the investigative nature of these subgroup analyses, future research should seek to replicate these effects in larger samples before drawing firm conclusions. See SI 10 in the online supplemental materials for the full set of adjusted *p* values.

#### Rerunning Models for SDQ Total Difficulties Without Controlling for Baseline YARC

In all analyses for the secondary outcome, SDQ total difficulties, we included baseline YARC scores as a control variable to account for preexisting differences between students in the two trial arms, as SDQ was not collected at baseline. While the absence of baseline SDQ data is a limitation, adjusting for YARC scores is preferable to relying solely on randomization to control for potential baseline differences.

As a robustness check, we compared model estimates with and without baseline YARC to evaluate the impact of this adjustment (see SI 11 in the online supplemental materials). Overall, including baseline YARC did not meaningfully change the overall treatment effects. However, in some subgroups—such as students with EAL and those from neighborhoods with varying levels of disadvantage (measured by IDACI)—adjusting for baseline YARC strengthened the evidence for differential treatment effects.

## Discussion

A nurturing, engaging, and supportive home environment is essential for children’s growth (Toth et al., 2020). Activities like reading together, introducing new words and phrases, providing structured guidance as children become more independent, and fostering warm, responsive relationships are associated with improved developmental outcomes at school entry (Bornstein et al., 2008; Bradley & Corwyn, 2005; Hart et al., 1997). Additionally, having access to resources like books and toys, combined with the quality of both implicit and explicit educational support from parents and caregivers, contributes to a rich home learning environment (E. Melhuish, 2010).

There is substantial evidence that parenting programs, in which a trained facilitator either visits families at home or parents attend group sessions with other caregivers over a set period, are effective in improving parenting skills and fostering supportive environments for children’s development, often leading to positive outcomes for both parents and children (Leijten et al., 2018; Mol et al., 2008; Sanders et al., 2014). However, such programs tend to be resource-intensive and costly, making it difficult to scale them for widespread use.

Less intensive programs that provide parents with accessible information and simple strategies without requiring a set time commitment are a promising alternative, potentially engaging parents who may not participate in more structured programs. While the universality of such programs is appealing, it is important to assess their overall impact, and more specifically their impact on different groups, particularly those facing challenges like socioeconomic disadvantage or language barriers. Understanding who benefits the most from such initiatives, and why, is crucial in times of constrained school budgets, where parental engagement programs are often the first to face cuts.

In this secondary data analysis, we examined the efficacy of a low intensity, text message intervention developed for parents of reception students (aged 4–5 years) in England. Parents received three text messages a week with tips and activities to do at home with their young children, all aimed at improving language, literacy, numeracy, and socioemotional skills.

The RCT faced substantial attrition because of the COVID-19 pandemic; however, the final sample size (*n* = 753) remained comparable to the original U.S. study (*n* = 823; York et al., 2019). Given this similarity, we deemed it worthwhile to proceed with the analysis despite the high level of attrition, as the sample still allowed for meaningful examination of the intervention’s effects and provided valuable insights into the replicability of the original findings.

### Analysis of Impact: Intervention Effects

Our findings indicate that the Tips by Text program did not produce significant improvements in reading-related outcomes or socioemotional well-being for students in the treatment group. Given the positive effects for low achievers reported in the original study (York et al., 2019), we had planned additional analyses to examine whether the program might have been particularly beneficial for low achievers, splitting the sample into low and high achievers based on baseline YARC scores. However, unlike the U.S. study, we found no evidence that the intervention benefited lower-achieving students nor did we observe a significant Treatment × Achievement interaction effect.

### Analysis of Differential Effect by SES

For our second set of analyses, we hypothesized that FSM-eligible students whose parents received Tips by Text would outperform those in the control group on reading-related outcomes and socioemotional well-being. We also hypothesized that students in more disadvantaged neighborhoods who received the program would show greater improvements in reading-related and socioemotional skills compared to those in the control group. Text message interventions have shown promise in improving academic outcomes for low-income students though primarily in older age groups (Avery et al., 2021; Bergman & Chan, 2021). Therefore, it is important to assess their impact on low SES populations and to incorporate multiple measures of SES for a more comprehensive evaluation.

For FSM-eligible students, we found no evidence that the program improved reading-related outcomes compared to the control group. Similarly, our analysis of its impact on SDQ total difficulties did not yield statistically significant results. The analysis of neighborhood disadvantage, measured using IDACI scores, also produced nonsignificant results for reading-related outcomes. For SDQ total difficulties, we found no clear evidence that the intervention influenced socioemotional difficulties either overall or across different levels of socioeconomic disadvantage. While there was a suggestion that students from more disadvantaged neighborhoods experienced greater socioemotional difficulties following the intervention, this trend was not statistically robust.

One possible explanation for these findings is the distribution of neighborhood deprivation within the sample. Less than 5% of students (179 out of 3,649) had IDACI scores above 0.5, indicating that only a small proportion came from highly deprived areas. The median IDACI score among those with baseline YARC assessments was 0.27, suggesting that, in terms of neighborhood deprivation, this was not a highly disadvantaged sample. This is somewhat unexpected given that nearly 30% of students (1,054 out of 3,649) were eligible for FSM, revealing a discrepancy between individual economic disadvantage (FSM eligibility) and area-level deprivation (IDACI scores). This mismatch suggests that the IDACI analysis may not have fully captured the most socioeconomically vulnerable students, potentially limiting the ability to detect meaningful intervention effects. IDACI is derived from the index of multiple deprivation, a composite measure of relative deprivation across small geographical areas, based on income, employment, education, health, crime, barriers to housing and services, and the living environment. Higher scores indicate greater deprivation. Some researchers have suggested that index of multiple deprivation-based measures, including IDACI, may be too broad to reliably predict individual differences in outcomes, particularly in younger age groups (Kneale & Lupton, 2010).

### Analysis of Differential Effects by Other Challenges

We had hypothesized that this type of program would be beneficial for EAL students. The intentional use of plain, accessible English in the text messages could have meant that parents with lower literacy levels or those whose first language was not English could engage with the program more easily. Text messages may effectively support parents who want to engage in their children’s development but may face barriers to participating in traditional parenting programs. For example, parents who are not confident in their language skills may be reluctant to join group-based programs or may find pamphlets or emails overwhelming. The concise nature of the text messages, limited to 160 characters and written in plain English, may have helped overcome some of these accessibility barriers for EAL parents who want to engage. Indeed, for EAL students, the intervention appeared to support improvements in reading-related skills, with results suggesting a trend toward greater gains in this domain. However, after adjusting for multiple comparisons, this effect was no longer statistically significant. In contrast, we found no evidence that the intervention reduced socioemotional difficulties among EAL students. Further research is needed to determine whether the small trend in our data only prior to correction for multiple comparisons will replicate and whether it extends to larger EAL populations. If so, future studies should also examine potential mechanisms, such as increased parental engagement, greater language exposure at home, or improved communication between parents and schools, that may mediate these effects.

For children with SEN, establishing clear directional hypotheses was more challenging. While delivering straightforward, engaging activities via text message may be beneficial, the program could also induce stress if not properly tailored to the child’s specific needs. Our results suggest that the intervention did not meaningfully affect reading-related outcomes or socioemotional well-being for this group. Given the complexity of supporting children with SEN, this result is perhaps unsurprising. However, we considered it important to examine this subgroup, as the intervention provided parents with additional support and included them in a program being rolled out to all students, which may have had indirect effects on the home learning environment and in turn, their children’s skills. More tailored interventions that offer direct, structured support specifically designed for students with SEN may be more effective in addressing their unique needs.

Overall, the intervention did not produce significant effects on the primary or secondary outcomes nor did it differentially impact low- versus high-achieving students or those experiencing socioeconomic (FSM) or neighborhood (IDACI) disadvantage. These null results suggest that, as implemented, the program did not meaningfully shift reading-related skills or socioemotional outcomes in this sample. The results for students with EAL showed some promise; yet following adjustment for multiple comparisons, the effect was no longer statistically significant. Given this and the small sample size (*n* = 68), further research is warranted to determine if this effect is genuine. Students with SEN showed no meaningful differences between treatment and control groups, suggesting that more tailored support may be necessary for this subgroup.

Taken together, our findings across these three sets of analysis suggest that while the intervention was well intentioned, it did not produce measurable improvements in reading-related skills or socioemotional outcomes. The absence of differential effects by achievement level, socioeconomic disadvantage, or other challenges (EAL and SEN) may suggest that low-intensity approaches may not be sufficient to drive improvements in these domains. This could be because of the “intention-action” gap, which refers to situations where individuals have the intention to engage in a particular behavior but fail to follow-through (Sheeran & Webb, 2016). In the context of parenting, even when parents intend to engage in activities that support their child’s development, competing demands or everyday challenges may prevent them from doing so. Strategies such as “implementation intentions”—which involve forming concrete plans that specify when, where, and how an action will be carried out—could help bridge this gap (Gollwitzer, 1999). By encouraging parents to set specific cues for action (e.g., “After dinner, I will spend 10 min reading with my child”), these techniques may increase follow-through and translate good intentions into meaningful behaviors that promote healthy child development.

Another possible explanation for the lack of significant effects is the choice of messenger used to deliver the intervention. The text messages were signed off with the student’s school name, an approach that was assumed to enhance credibility and engagement. However, this assumption warrants further scrutiny. While schools are central to children’s education, parents may perceive school-affiliated messages as directive rather than supportive or they may associate them with administrative rather than developmental guidance. As a result, the intervention may not have resonated as intended. Future research should explore whether the effectiveness of text message-based interventions is influenced by the perceived authority or relatability of the messenger. For instance, messages endorsed by child development experts may be seen as more evidence based, while those from community support officers could feel more approachable. Testing different messengers such as educators, developmental psychologists, or trusted community figures could provide valuable insights into whether variations in perceived expertise, trust, and relatability impact parental engagement and follow-through. Additionally, future studies should examine whether adaptations such as supplementary resources, alternative communication mediums (e.g., voice notes on WhatsApp), or greater tailoring to specific learner needs could further enhance the effectiveness of such interventions. Understanding these dynamics may be key to optimizing the effectiveness of similar interventions in the future.

Finally, it is important to acknowledge that this trial took place during the COVID-19 pandemic, which led to a nearly 70% loss of postintervention data. This substantial attrition reduced the study’s power to detect effects, particularly for smaller subgroups. A retrial of the Tips by Text program is currently underway (Education Endowment Foundation, 2024), with results expected in 2026, which should provide more robust evidence on the program’s effectiveness.

### Limitations and Future Directions

This original RCT and by extension, the secondary analyses described in this study, had several limitations. First, the primary outcome measure, the YARC, proved to be too challenging for the age group involved. Many children scored close to zero on the baseline assessment (see SI 12 in the online supplemental materials for histograms), indicating that the tasks were generally too difficult for a significant portion of pupils. This resulted in a floor effect where the low scores limited the ability to detect meaningful differences in performance. It is difficult to find measures that are comprehensive and can be delivered at scale. Measures that are more age appropriate for 4- to 5-year-olds should be considered in future. Potential options may include the LanguageScreen (Hulme et al., 2024; West et al., 2021) or the PALS (Invernizzi et al., 2004), which was used in the original study (York et al., 2019).

Second, although we did not collect data on potential spillover, it is likely that some occurred. During intervention piloting, we asked parents whether they would share the content with friends or other parents in their child’s class. The responses were mixed. Some parents and teachers suggested that parents might physically show the messages to each other during pick-up and drop-off times, while others felt the content was specific to their child and were less inclined to share it. Conducting a cluster RCT, where schools rather than parents are randomly assigned to the treatment or control group, could help avoid the risk of contamination of the control group.

Third, the passive (“business as usual”) control used in the RCT posed a limitation, as it did not provide an active comparison, making it difficult to determine if any observed effects are attributable to the intervention itself or simply the result of increased engagement. During the RCT’s planning phase, we considered sending messages to the control group but ultimately decided against it, as developing enough neutral content for a 12-month program within the available time frame for intervention development was not feasible. Ideally, a three-arm RCT, comparing the intervention with both passive and active control groups, would better isolate the impact of the content itself from the effects of engagement through text messages.

Fourth, during the pilots, several parents mentioned that the activities were too easy for their children. We intentionally selected activities targeting the lower end of the achievement distribution to ensure that those who might benefit most from additional support could engage with the content. Ideally, activities would be tailored for both low and high achievers, which could easily be done using baseline assessment data to assign more challenging activities to pupils scoring above a predefined cutoff. However, there is a potential risk that some parents might feel stigmatized if their child is not in the higher-achieving group. This would largely depend on parents actively comparing the content however, which seems unlikely. A differentiated program has already been tested in the United States with promising results—pupils whose parents received a differentiated and personalized program were 63% more likely to read at a higher level compared to those receiving a more general, non-differentiated program. Moreover, parents in the differentiated group reported higher engagement in literacy activities with their children (Doss et al., 2019).

Finally, throughout the trial, the researchers were responsible for sending the text messages to parents. To better simulate real-world conditions, future studies should explore how teachers and school administrators could manage the text message distribution themselves. Most schools already have texting platforms, meaning the infrastructure for delivering an intervention like Tips by Text is already in place. Future research should investigate whether and how the intervention’s impact changes when delivered by teachers rather than researchers. Although much of the messaging can be automated, implementing such programs still requires staff time to monitor the platform daily for safeguarding purposes, ensure message delivery, and follow-up with parents if messages go undelivered for an extended period.

## Conclusion

Digital interventions for parents have expanded rapidly in recent years, with thousands of apps available to support child development (Fedorychak, 2024). While apps offer advantages such as gamification and engaging content, they also present challenges, including low uptake, inconsistent use, and the risk of excluding parents who are less digitally proficient (Zhang & Livingstone, 2019). In contrast, text messages may provide a simple, accessible, and widely used method for delivering parenting support, particularly when integrated into existing school communication systems.

The aim of this study was to replicate the primary impact analysis conducted by the original RCT evaluators of Tips by Text in the United Kingdom and expand upon it by investigating whether the intervention had varying effects on children experiencing different challenges, such as socioeconomic disadvantage, language barriers, and SEN. In contrast with findings in a comparably sized U.S.-based sample (York et al., 2019), our results revealed no significant improvements in reading-related skills or socioemotional outcomes nor any notable differences in how these subgroups responded to the program. The substantial attrition rate resulted in underpowered analyses, reducing our ability to reliably detect potential effects.

A large-scale retrial of the Tips by Text program is currently underway, with results expected in 2026 (Education Endowment Foundation, 2024), which should provide more definitive evidence of its impact. Future research should also consider factors such as the role of the messenger, message content, and parental engagement strategies to optimize effectiveness. Despite the null findings in this study, text message-based interventions remain a promising area for further research on supporting early childhood development, particularly if adapted to better meet the needs of diverse families. Continued investigation is needed to assess their full potential.

## Supplementary Material

10.1037/edu0000973.supp

## Figures and Tables

**Table 1 tbl1:** Sample Sizes Across Three Time Points

Sample characteristics	*N*	Pretrial (anticipated)				Randomization				Analysis			
		Full	FSM	EAL	SEN	Full	FSM	EAL	SEN	Full	FSM	EAL	SEN
Number of settings	Total	105	105	105	105	109	109	109	109	38	34	21	28
Number of pupils	Treat.	1,365	525	315	157	1,325	521	170	235	369	119	29	35
	Cont.	1,365	525	315	158	1,322	533	179	222	384	108	39	33
	Total	2,730	1,050	630	315	2,647	1,054	349	457	753	227	68	68
*Note.* FSM = free school meal; EAL = English as an additional language; SEN = special educational needs; Treat. = treatment; Cont. = control.													

**Table 2 tbl2:** Statistical Power

Statistic	Level	Pretrial (anticipated)				Randomization				Analysis			
		Full	FSM	EAL	SEN	Full	FSM	EAL	SEN	Full	FSM	EAL	SEN
Minimum detectable effect size		0.10	0.14	0.17	0.24	0.10	0.13	0.23	0.20	0.14	0.26	0.51	0.53
Pretest/posttest correlations	Level 1 (pupil)	.40	.40	.40	.40	.40	.40	.40	.40	.50	.50	.50	.50
Intercluster correlations	Level 2 (school)	.10	.10	.10	.10	.10	.10	.10	.10	.06	.06	.06	.06
Alpha level (α)		.05	.05	.05	.05	.05	.05	.05	.05	.05	.05	.05	.05
Power (1−β)		0.80	0.80	0.80	0.80	0.80	0.80	0.80	0.80	0.80	0.80	0.80	0.80
One-sided or two-sided?		2	2	2	2	2	2	2	2	2	2	2	2
Average cluster size		26	10	6	3	24	10	3	4	20	7	3	2
*Note.* All power calculations were conducted in PowerUp! (Dong & Maynard, 2013). Calculations are based on one-class per school. In schools with more than one reception class, the class to be assessed was chosen randomly. The analysis sample consists of pupils for whom postintervention YARC data were successfully collected. Further information on how these estimates were calculated is in SI 4 in the online supplemental materials. FSM = free school meal; EAL = English as an additional language; SEN = special educational needs; YARC = York Assessment of Reading Comprehension.													

**Table 3 tbl3:** Descriptive Statistics for the Key Study Variables

Measure	Randomized sample					Assessed sample					Analysis sample				
	*n*	*M* (*SD*)	*Mdn*	Min.	Max.	*n*	*M* (*SD*)	*Mdn*	Min.^a^	Max.^a^	*n*	*M* (*SD*)	*Mdn*	Min.^a^	Max.^a^
YARC (overall)^b^						2,389	0.209^c^ (0.187)	0.174	0	1	781	0.657^d^ (0.184)	0.675	0	1
Cont.						1,204	0.197	0.167	0	0.958	393	0.653 (0.188)	0.675	0.036	1
Treat.						1,185	0.204	0.167	0	0.917	388	0.661 (0.180)	0.689	0.000	0.988
IDACI (overall)	3,649	0.269 (0.141)	0.272	0.010	0.62	2,384	0.270 (0.139)	0.273	0.010	0.602	781	0.273 (0.132)	0.279	0.019	0.567
Cont.	1,824	0.267 (0.140)	0.268	0.010	0.605	1,201	0.269 (0.139)	0.268	0.011	0.591	393	0.273 (0.135)	0.277	0.022	0.558
Treat.	1,825	0.270 (0.142)	0.276	0.010	0.606	1,183	0.271 (0.138)	0.275	0.010	0.583	388	0.273 (0.130)	0.279	0.025	0.546
Age in months (overall)	3,639	56.56 (3.56)	57	51	65.9	2,390	54.808 (3.693)	55	51	65.9	781	69.638 (3.616)	70	64	76.4
Cont.	1,819	56.54 (3.55)	57	51	64	1,205	54.799 (3.663)	55	51	64	393	69.746 (3.621)	70	64	75.3
Treat.	1,820	56.58 (3.57)	57	51	65.4	1,185	54.817 (3.726)	55	51	65.4	388	69.528 (3.613)	70	64	76.1
Gender (overall)	50.21% male (*n* = 1,832/3,639)					49.87% male (*n* = 1,192/2,390)					51.22% male (*n* = 400/781)				
Cont.	50.08% male (*n* = 911/1,819)					51.24% (*n* = 617/1,204)					51.65% male (*n* = 203/393)				
Treat.	50.60% male (*n* = 921/1,819)					48.56% (*n* = 575/1,184)					50.77% male (*n* = 197/388)				
FSM (overall)	28.88% FSM-eligible (*n* = 1,054/3,649)					27.87% FSM eligible (*n* = 666/2,390)					30.15% FSM eligible (*n* = 227/753)				
Cont.	29.22% FSM-eligible (*n* = 533/1,824)					27.88% FSM eligible (*n* = 336/1,205)					47.58% (*n* = 108/227)				
Treat.	28.55% FSM-eligible (*n* = 521/1,825)					27.85% FSM eligible (*n* = 330/1,185)					52.42% (*n* = 119/227)				
EAL (overall)	9.56% with EAL (*n* = 349/3,649)					7.62% with EAL (*n* = 182/2,390)					9.03% (*n* = 68/753)				
Cont.	9.81% with EAL (*n* = 179/1,824)					8.30% with EAL (*n* = 100/1,205)					57.35% (*n* = 39/68)				
Treat.	9.32% with EAL (*n* = 170/1,825)					6.92% with EAL (*n* = 82/1,185)					42.65% (*n* = 29/68)				
SEN (overall)	12.52% with SEN (*n* = 457/3,649)					10.75% with SEN (*n* = 257/2,390)					9.03% (*n* = 68/753)				
Cont.	12.17% with SEN (*n* = 222/1,824)					10.71% with SEN (*n* = 129/1,205)					48.53% (*n* = 33/68)				
Treat.	12.88% with SEN (*n* = 235/1,825)					10.80% with SEN (*n* = 128/1,185)					51.47% (*n* = 35/68)				
*Note.* Min. = minimum; Max. = maximum; YARC = York Assessment of Reading Comprehension; Cont. = control; Treat. = treatment; IDACI = Income Deprivation Affecting Children Index; FSM = free school meal; EAL = English as an additional language; SEN = special educational needs.															
^a^ The minimum and maximum values for IDACI and age in months are derived from the National Pupil Database. To protect participant anonymity, these values represent the average of the 10 smallest and 10 largest observations rather than individual minimum and maximum values. ^b^ The summed raw scores were normalized using Min–Max scaling, where each value was transformed to a 0–1 range by subtracting the minimum observed value and dividing by the range (maximum−minimum). ^c^ The YARC at baseline included two subscales only. ^d^ The YARC at end line included all four subscales.															

**Table 4 tbl4:** Impact Analysis: The Effect of the Treatment on the Primary and Secondary Outcomes

Outcome	Unadjusted means^a^						
	Control		Treatment		Effect size		
	*n*	*M* (*SD*)	*n*	*M* (*SD*)	Total *N*	Hedge’s *g* [95% CI]	*p*
YARC	384	−0.022 (1.011)	369	0.023 (0.989)	753	0.020 [−0.094, 0.134]	.730
Total difficulties	517	6.936 (5.534)	520	7.035 (5.662)	1,037	0.086 [−0.023, 0.196]	.123
Internalizing	517	2.512 (2.798)	520	2.568 (2.737)	1,037	0.067 [−0.046, 0.179]	.248
Externalizing	517	4.423 (4.004)	520	4.467 (4.211)	1,037	0.07 [−0.040, 0.182]	.208
Prosocial	517	7.218 (2.649)	520	7.361 (2.523)	1,037	−0.019 [−0.124, 0.085]	.714
*Note.* CI = confidence interval; YARC = York Assessment of Reading Comprehension.							
^a^ Unadjusted means represent the raw, observed averages, and standard deviations for the treatment and control groups, calculated directly from the data without accounting for covariates or clustering effects.							

**Table 5 tbl5:** Treatment Effects by Baseline Achievement Level (High vs. Low Achievers)

Outcome	Unadjusted means						
	Control		Treatment		Effect size		
	*n*	*M* (*SD*)	*n*	*M* (*SD*)	Total *N*	Hedge’s *g* [95% CI]	*p*
YARC							
Low achievers (below median on baseline YARC)	194	−0.468 (1.009)	181	−0.367 (1.005)	375	0.101 [−0.102, 0.303]	.331
High achievers (above median on baseline YARC)	190	0.477 (0.732)	188	0.457 (0.746)	378	0.028 [−0.229, 0.174]	.787
SDQ total difficulties							
Low achievers (below median on baseline YARC)	268	8.093 (5.528)	237	8.228 (5.455)	505	0.024 [−0.150, 0.199]	.784
High achievers (above median on baseline YARC)	249	5.136 (4.913)	283	5.731 (5.485)	532	−0.114 [−0.057, 0.284]	.190
*Note.* CI = confidence interval; YARC = York Assessment of Reading Comprehension; SDQ = Strengths and Difficulties Questionnaire.							

**Table 6 tbl6:** Interaction Effects of Treatment and Baseline Achievement

Measure	β	*SE*	*t*	*p*	*n*
YARC					
Treatment	−0.035	0.081	−0.430	.667	753
Low achievers (below median on baseline YARC)	−0.225	0.109	−2.056	.04	753
Treatment × Low Achievers	0.114	0.116	0.980	.32	753
SDQ total difficulties					
Treatment	0.675	0.432	1.561	.119	1,037
Low achievers (below median on baseline YARC)	1.311	0.58	2.261	.024	1,037
Treatment × Low Achievers	−0.404	0.622	−0.650	.516	1,037
*Note.* YARC = York Assessment of Reading Comprehension; SDQ = Strengths and Difficulties Questionnaire.					

**Table 7 tbl7:** Intervention Effects for FSM Eligible and Noneligible Students on YARC Scores

Outcome	Unadjusted means						
	Control		Treatment		Effect size		
	*n*	*M* (*SE*)	*n*	*M* (*SD*)	Total *N*	Hedge’s *g* [95% CI]	*p*
YARC							
FSM eligible	108	−0.354 (0.999)	119	−0.174 (1.069)	227	0.093 [−0.135, 0.322]	.425
FSM noneligible	276	0.137 (0.968)	250	0.160 (0.907)	526	0.017 [−0.118, 0.152]	.807
SDQ total difficulties							
FSM eligible	147	7.605 (5.687)	162	7.253 (5.510)	309	−0.041 [−0.256, 0.173]	.707
FSM noneligible	369	6.287 (5.306)	358	6.70 (5.648)	727	0.130 [−0.002, 0.262]	.054
*Note.* FSM = free school meal; YARC = York Assessment of Reading Comprehension; CI = confidence interval; SDQ = Strengths and Difficulties Questionnaire.							

**Table 8 tbl8:** Interaction Effects of Treatment and FSM Eligibility

Measure	β	*SE*	*t*	*p*	*n*
YARC					
Treatment	0.013	0.069	0.192	.848	753
FSM eligible	−0.268	0.093	−2.889	.004	753
Treatment × FSM Eligible	0.058	0.127	0.452	.652	753
SDQ total difficulties					
Treatment	0.721	0.370	1.949	.052	1,036
FSM eligible	1.30	0.502	2.579	.010	1,036
Treatment × FSM Eligible	−0.962	0.687	−1.399	.162	1,036
*Note.* FSM = free school meal; YARC = York Assessment of Reading Comprehension; SDQ = Strengths and Difficulties Questionnaire.					

**Table 9 tbl9:** Interaction Effects of Treatment and IDACI

Measure	β	*SE*	*t*	*p*	*n*
YARC					
Treatment	−0.149	0.132	−1.136	.256	753
IDACI	−0.797	0.331	−2.405	.016	753
Treatment × IDACI	0.621	0.434	1.432	.152	753
SDQ total difficulties					
Treatment	−0.977	0.796	−1.227	.220	931
IDACI	−1.568	1.891	−0.829	.407	931
Treatment × IDACI	4.899	2.476	1.979	.048	931
*Note.* IDACI = Income Deprivation Affecting Children Index; YARC = York Assessment of Reading Comprehension; SDQ = Strengths and Difficulties Questionnaire.					

**Table 10 tbl10:** Intervention Effects of Treatment and EAL on YARC and SDQ

Outcome	Unadjusted means						
	Control		Treatment		Effect size		
	*n*	*M* (*SD*)	*n*	*M* (*SD*)	Total *N*	Hedges’ *g* [95% CI]	*p*
YARC							
EAL	39	−0.231 (1.126)	29	0.191 (0.817)	68	0.375 [0.008, 0.742]	.045
Non-EAL	345	0.025 (0.984)	340	0.041 (0.986)	685	−0.010 [−0.130, 0.109]	.864
SDQ total difficulties							
EAL	46	7.435 (5.445)	35	6.029 (5.009)	81	−0.379 [−0.791, 0.033]	.072
Non-EAL	470	6.587 (5.443)	485	6.930 (5.647)	955	0.106 [−0.009, 0.220]	.071
*Note.* EAL = English as an additional language; YARC = York Assessment of Reading Comprehension; SDQ = Strengths and Difficulties Questionnaire; CI = confidence interval.							

**Table 11 tbl11:** Interaction Effects of Treatment and EAL on YARC

Measure	β	*SE*	*t*	*p*	*n*
YARC					
Treatment	−0.012	0.060	−0.192	.848	753
EAL	−0.027	0.139	−0.195	.845	753
Treatment × EAL	0.404	0.203	1.988	.047	753
SDQ total difficulties					
Treatment	0.591	0.322	1.834	.067	1,036
EAL	−0.005	0.818	−0.006	.995	1,036
Treatment × EAL	−1.843	1.182	−1.559	.119	1,036
*Note.* EAL = English as an additional Language; YARC = York Assessment of Reading Comprehension; SDQ = Strengths and Difficulties Questionnaire.					

**Table 12 tbl12:** Intervention Effects of Treatment and SEN on YARC

Outcome	Unadjusted means						
	Control		Treatment		Effect size		
	*n*	*M* (*SD*)	*n*	*M* (*SD*)	Total *N*	Hedge’s *g* [95% CI]	*p*
YARC							
SEN	33	−1.101 (0.918)	35	−1.028 (1.241)	68	−0.010 [−0.427, 0.408]	.964
Non-SEN	351	0.103 (0.945)	334	0.166 (0.868)	685	0.037 [−0.084, 0.158]	.552
SDQ total difficulties							
SEN	63	11.190 (6.133)	61	10.410 (5.841)	124	−0.137 [−0.492, 0.219]	.452
Non-SEN	454	6.042 (5.033)	459	6.399 (5.409)	913	0.107 [−0.009, 0.223]	.070
*Note.* SEN = special educational needs; YARC = York Assessment of Reading Comprehension; CI = confidence interval; SDQ = Strengths and Difficulties Questionnaire.							

**Table 13 tbl13:** Interaction Effects of Treatment and SEN on YARC

Measure	β	*SE*	*t*	*p*	*n*
YARC					
Treatment	0.038	0.058	0.653	.514	753
SEN	−0.734	0.145	−5.064	.000	753
Treatment × SEN	−0.075	0.196	−0.382	.703	753
SDQ total difficulties					
Treatment	0.564	0.323	1.748	.081	1,036
SEN	3.985	0.676	5.893	.000	1,036
Treatment × SEN	−1.061	0.940	−1.128	.260	1,036
*Note.* SEN = special educational needs; YARC = York Assessment of Reading Comprehension; SDQ = Strengths and Difficulties Questionnaire.					

## Footnotes

1 A children’s centre is a community-based facility that provides integrated services, such as early childhood education, family support, health care, and parenting advice, to support the development and well-being of young children and their families.

2 Reception year in the United Kingdom is equivalent to kindergarten in the United States; it is the first year of primary school for children aged 4–5, focusing on foundational skills in literacy, numeracy, and social development to prepare them for Year 1.

3 The original trial was registered at https://www.controlled-trials.com and is available at https://doi.org/10.1186/ISRCTN17575452.
