# Supplementary material for: Supporting Parents to Support Children: A U.K. Randomized Controlled Trial Testing a Text Message Intervention to Cultivate the Home Learning Environment
Source: J Educ Psychol. 2025 Aug 28;118(1):12–34. doi: 10.1037/edu0000973 (PMC12680085; doi:10.1037/edu0000973)

**Supporting parents to support children. A UK Randomised Controlled Trial testing a text-message intervention to cultivate the home learning environment. Supplementary Information (SI)**

### **SI 1: Exemplar text messages**

Table 14: Selection of text messages sent to parents

| Theme | FACT | TIP | GROWTH |
| --- | --- | --- | --- |
| Literacy - vocabulary | FACT: Knowing lots of words is important for reading, writing and talking to others. Children who know many words can express themselves better! | TIP: As you do everyday tasks around the house, explain what you're doing to {{child first name}}. For example, tell {{child him-her}} that you WASH dishes so as to CLEAN them. To WASH is to CLEAN. | GROWTH: Keep explaining new words as you come across them. Ask questions about the words: "What else do we WASH or CLEAN?" (hair, teeth). "What do we use to wash or clean them?" (shampoo, toothpaste). |
| Math - geometry | FACT: Shapes are all around us! Pointing out shapes and asking children questions about them can help build their math skills. | TIP: Look for shapes in your home. Point and say: "The window is a rectangle." Then ask {{child first name}}: "What shape are the plates?" (circles) | GROWTH: Keep pointing out shapes. Make it a game! Who can find a circle, square, rectangle, and triangle? Are they big or small? You could also play with 3D shapes, like cubes (box) or spheres (apple). |
| Socioemotional understanding | FACT: Play time is not only fun, it also allows children to be active, discover, explore, imagine, solve problems and test new ideas. | TIP: Join {{child first name}} when {{child he-she}} is playing today! Ask {{child him-her}} what {{child his-her}} teddy is thinking about, or what teddy likes to eat. | GROWTH: Keep playing together! Use activities like cooking to teach teamwork and cooperation - work together to make dinner or a cake, taking it in turns to stir and add ingredients. |

### **SI 2: Theory of Change**

**Problem statement:** Many parenting programs place significant demands on parents at a time in their lives when they may be feeling stretched by the demands of having a young family. Programs that provide parents with flexibility and involve activities they can be easily incorporated into their daily routines could help overcome such constraints.

Table 15: Theory of Change

| **Inputs** | **Activities** | **Outputs** | **Short-term Outcomes** | **Long-term Outcomes** |
| --- | --- | --- | --- | --- |
| Student and parent participants from 109 schools from the North- East.  Student and parent details (names, pronouns, telephone numbers).  Time needed to send out weekly messages.  Time needed for monitoring parent responses and escalating any safeguarding concerns.  Time needed to update parent telephone numbers as needed. | Parents are sent three messages a week for 12 months.  There are three types of messages:  - FACT messages inform and motivate parents by emphasizing the importance of specific skills.  - TIP messages provide brief, simple, and targeted activities for parents to integrate into existing family routines.  - GROWTH messages offer encouragement, reinforcement, and expand upon the "TIP" texts.  Ongoing support to schools is provided via email. | Parents incorporate the activities into their daily routines.  Parents understand the importance of developing different skills in their children.  Parents feel confident in coming up with similar activities by themselves. | Increased parental confidence in incorporating new activities with their children.  Enhanced understanding among parents of the EYFS-derived activities that support their children's development.  Increased parental engagement in their children's learning process.  More frequent learning opportunities for children at home. | Enhanced HLE* parents report increased warmth, consistency, and frequency of activities with their children.  Improved HLE results in better child outcomes in literacy, numeracy, language, communication, and social development.  Children's increased abilities upon starting school lead to improved outcomes later in their education, setting the foundation for a more successful school experience.  Strengthened relationships between parents and schools. |

* HLE is an abbreviation for the home learning environment.

### **SI 3: CONSORT Participant Flow Diagram**

Figure 3: CONSORT Flow Diagram - Tips by Text RCT


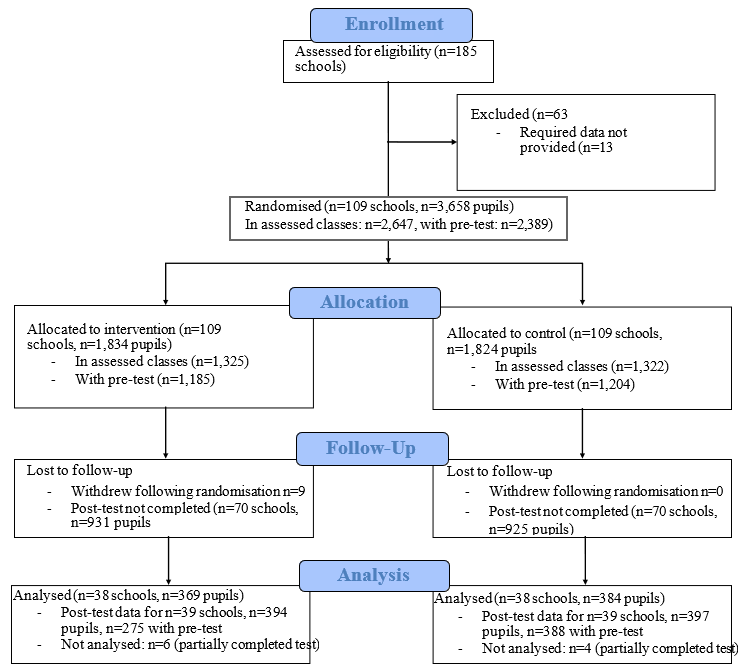


- Note, parents and not students were randomized to avoid twins being allocated to both the treatment and control groups.
- The analysis sample includes those with fully completed assessments at baseline and endline. Those with only partially completed assessments were omitted.

### **SI 4: Additional information on power calculations**

**Power calculations for additional variables – EAL and SEN**

In the original RCT, the impact of the intervention on sub-groups other than those receiving free school meals (FSM) was not prioritized by the funders or appointed evaluators. These additional variables were collected retrospectively from the National Pupil Database. Consequently, we conducted a post-hoc power analysis and used national statistics to calculate pre-trial power. Detailed calculations are provided below. Post hoc power calculations for the sample at the point of randomization and at the end of the trial (i.e., the analysis sample) will be calculated as part of the analysis for this paper.

**English as an additional language (pre-trial)**

*Calculating the number of EAL pupils per class*

- EAL pupils in state funded primaries 2019/20: 1,002,387
- Overall number of pupils in state funded primaries 2019/20: 4,714,772
- 1,002,387 / 4,714,772 = 0.2126056
- N=26 per class (assumption by appointed evaluators for the original trial)
- 26 * 0.2126056 = 5.527746, rounded to 6 EAL per class
- 105 schools * 6 pupils = 630, randomly allocated to treatment (n=315) and control (n=15)
- MDES = 0.17 (calculated using PowerUp!)

*Calculating the number of SEN pupils per class*

- SEN pupils in state funded primaries 2019/20: 602,616
- Overall number of pupils in state funded primaries 2019/20: 4,714,772
- 602,616 / 4,714,772 = 0.1278145
- N=26 per class (assumption by appointed evaluators for the original trial)
- 26 * 0.1278145 = 3.323177, rounded to 3 SEN per class
- 105 schools * 3 pupils per class = 315, randomly allocated to treatment (n=157) and control (n=158)
- MDES = 0.235 (calculated using PowerUp!)

### **SI 5: Checking for balance across the treatment and control groups**

Table 16: YARC scores at baseline in each sub-group

| **Trial Arm** | **N** | **Mean (SD)*** | **Median** | **Min** | **Max** |
| --- | --- | --- | --- | --- | --- |
| **Overall sample** | | | | | |
| *Total* | 2,389 | 0.201 (0.180) | 0.167 | 0 | 0.958 |
| *Cont.* | 1,204 | 0.197 (0.174) | 0.167 | 0 | 0.958 |
| *Treat.* | 1,185 | 0.204 (0.184) | 0.167 | 0 | 0.917 |
| **Sub-group: Free School Meals (FSM)** | | | | | |
| *Total* | 665 | 0.153 (0.154) | 0.125 | 0 | 0.792 |
| *Cont.* | 335 | 0.146 (0.137 | 0.125 | 0 | 0.625 |
| *Treat.* | 330 | 0.160 (0.169) | 0.125 | 0 | 0.792 |
| **Sub-group: English as an Additional Language** | | | | | |
| *Total* | 182 | 0.127 (0.171) | 0.042 | 0 | 0.833 |
| *Cont.* | 100 | 0.122 (0.153) | 0.083 | 0 | 0.667 |
| *Treat.* | 82 | 0.133 (0.191) | 0.042 | 0 | 0.833 |
| **Sub-group: Special Educational Needs (SEN)** | | | | | |
| *Total* | 257 | 0.100 (0.136) | 0.042 | 0 | 0.792 |
| *Cont.* | 129 | 0.111 (0.132) | 0.083 | 0 | 0.708 |
| *Treat.* | 128 | 0.089 (0.140) | 0.042 | 0 | 0.792 |
| **Sub-group: Neighborhood disadvantage – Income Deprivation Affecting Children Index (IDACI)** | | | | | |
| *Total* | 2,383 | 0.201 (0.180) | 0.167 | 0 | 0.958 |
| *Cont.* | 1,200 | 0.197 (0.174) | 0.167 | 0 | 0.958 |
| *Treat.* | 1,183 | 0.204 (0.185) | 0.167 | 0 | 0.917 |
|  |  |  |  |  |  |

*The above scores reflect the standardized scores.

Table 17: YARC scores: Analysis sample (post-intervention)

| **Trial Arm** | **N** | **Mean (SD)*** | **Median** | **Min** | **Max** |
| --- | --- | --- | --- | --- | --- |
| **Sub-group: Free School Meals (FSM)** | | | | | |
| *Cont.* | 108 | -0.354 (0.999) | -0.178 | -3.455 | 1.626 |
| *Treat.* | 119 | -0.174 (1.069) | -0.139 | -3.794 | 1.739 |
| **Sub-group: English as an Additional Language** | | | | | |
| *Cont.* | 39 | -0.231 (1.126) | -0.189 | -2.117 | 1.581 |
| *Treat.* | 29 | 0.191 (0.817) | 0.289 | -1.849 | 1.517 |
| **Sub-group: Special Educational Needs (SEN)** | | | | | |
| *Cont.* | 33 | -1.101 (0.918) | -1.308 | -3.455 | 1.154 |
| *Treat.* | 35 | -1.028 (1.241) | -1.065 | -3.794 | 1.681 |
| **Sub-group: Neighborhood disadvantage – Income Deprivation Affecting Children Index (IDACI)** | | | | | |
| *Cont.* | 384 | -0.001 (1.001) | 0.151 | -3.590 | 1.850 |
| *Treat.* | 369 | 0.052 (0.973) | 0.186 | -3.794 | 1.739 |
|  |  |  |  |  |  |

*The above scores reflect the standardized scores.

Table 18: SDQ Total Difficulties scores at endline broken down by sub-group

| **Trial Arm** | **N** | **Mean (SD)*** | **Median** | **Min** | **Max** |
| --- | --- | --- | --- | --- | --- |
| **Overall** | | | | | |
| *Total* | 1,124 | 6.986 (5.597) | 6 | 0 | 33 |
| *Cont.* | 559 | 6.936 (5.534) | 6 | 0 | 27 |
| *Treat.* | 565 | 7.035 (5.662) | 6 | 0 | 33 |
| **Sub-group: Free School Meals (FSM)** | | | | | |
| *Overall* | 342 | 7.684 (5.627) | 7 | 0 | 25 |
| *Cont.* | 160 | 7.963 (5.704) | 7 | 0 | 25 |
| *Treat.* | 182 | 7.440 (5.562) | 6 | 0 | 23 |
| **Sub-group: English as an Additional Language** | | | | | |
| *Overall* | 95 | 7.389 (5.288) | 6 | 0 | 21 |
| *Cont.* | 53 | 8.038 (5.470) | 7 | 0 | 20 |
| *Treat.* | 42 | 6.571 (4.993) | 5.5 | 0 | 21 |
| **Sub-group: Special Educational Needs (SEN)** | | | | | |
| *Overall* | 156 | 11.135 (5.946) | 11 | 0 | 26 |
| *Cont.* | 80 | 11.538 (6.008) | 11 | 1 | 25 |
| *Treat.* | 76 | 10.711 (5.890) | 10 | 0 | 26 |
| **Sub-group: Neighborhood disadvantage – Income Deprivation Affecting Children Index (IDACI)** | | | | | |
| *Overall* | 1,121 | 6.988 (5.602) | 6 | 0 | 33 |
| *Cont.* | 557 | 6.935 (5.541) | 6 | 0 | 27 |
| *Treat.* | 564 | 7.041(5.666) | 6 | 0 | 33 |
|  |  |  |  |  |  |

*The above scores reflect the standardized scores.

Table 19: SDQ Total Difficulties scores for the full sample broken down by sub-scale

| **Trial Arm** | **N** | **Mean (SD)*** | **Med.** | **Min.** | **Max.** | **Q1** | **Q3** | **IQR** |
| --- | --- | --- | --- | --- | --- | --- | --- | --- |
| **Total Difficulties** (sum of subscales 1-4 below; higher scores mean more difficulties) | | | | | | | | |
| Overall | 1,124 | 6.986 (5.597) | 6 | 0 | 33 | 2 | 10 | 8 |
| Treat. | 559 | 6.936 (5.534) | 6 | 0 | 27 | 2 | 10 | 8 |
| Cont. | 565 | 7.035 (5.662) | 6 | 0 | 33 | 2 | 11 | 9 |
| **1.Emotional Symptoms** (higher scores mean more emotional difficulties) | | | | | | | | |
| Overall | 1,124 | 1.367 (1.867) | 0.5 | 0 | 10 | 0 | 2 | 2 |
| Treat. | 559 | 1.335 (1.862) | 0 | 0 | 10 | 0 | 2 | 2 |
| Cont. | 565 | 1.400 (1.873) | 1 | 0 | 10 | 0 | 2 | 2 |
| **2.Conduct Problems** (higher scores mean more conduct problems) | | | | | | | | |
| Overall | 1,124 | 0.899 (1.491) | 0 | 0 | 10 | 0 | 1 | 1 |
| Cont. | 559 | 0.862 (1.409) | 0 | 0 | 8 | 0 | 1 | 1 |
| Treat. | 565 | 0.936 (1.569) | 0 | 0 | 10 | 0 | 1 | 1 |
| **3.Hyperactivity / inattention** (higher scores mean more difficulty with attention) | | | | | | | | |
| Overall | 1,124 | 3.546 (3.059) | 3 | 0 | 10 | 1 | 5 | 4 |
| Cont. | 559 | 3.562 (3.005) | 3 | 0 | 10 | 1 | 5 | 4 |
| Treat. | 565 | 3.531 (3.114) | 3 | 0 | 10 | 1 | 5 | 4 |
| **4.Peer Relation Problems** (higher scores mean more difficulty in social interactions) | | | | | | | | |
| Overall | 1,124 | 1.173 (1.586) | 0 | 0 | 8 | 0 | 2 | 2 |
| Cont. | 559 | 1.177 (1.599) | 0 | 0 | 8 | 0 | 2 | 2 |
| Treat. | 565 | 1.168 (1.574) | 0 | 0 | 8 | 0 | 2 | 2 |
| **5.Prosocial Behavior** (higher scores mean more prosocial behavior) | | | | | | | | |
| Overall | 1,124 | 7.290 (2.586) | 8 | 0 | 10 | 5 | 10 | 5 |
| Cont. | 559 | 7.218 (2.649) | 8 | 0 | 10 | 5 | 10 | 5 |
| Treat. | 565 | 7.361 (2.523) | 8 | 0 | 10 | 5 | 10 | 5 |
|  |  |  |  |  |  |  |  |  |

### **SI 6: Sample Characteristics**

Table 20: School Characteristics

| Variable | National (%) | School sample (n/N) | School sample (%) |
| --- | --- | --- | --- |
| **School Location** | | | |
| Missing | - | 0/109 | 0.00% |
| Rural hamlet and isolated dwellings | NA^1^ | 1/109 | 0.92% |
| Rural town and fringe | NA | 9/109 | 8.26% |
| Rural town and fringe in a sparse setting | NA |  | 0.92% |
| Urban city and town | NA | 47/109 | 43.12% |
| Urban city and town in a sparse setting | NA | 1/109 | 0.92% |
| Urban major conurbation | NA | 50/109 | 45.87% |
| **School Type** | | | |
| Missing | - | 0/109 | 0.00% |
| Academy converter | 25% | 32/109 | 29.36% |
| Academy sponsor led | 9% | 4/109 | 3.67% |
| Community school | 36% | 44/109 | 40.37% |
| Foundation school | 3% | 12/109 | 11.01% |
| Voluntary aided / controlled | 25% | 17/109 | 15.60% |
| **School Overall Effectiveness (Ofsted)** | | | |
| Missing | - | 5 | 4.59% |
| Outstanding | 17% | 13 | 12.50% |
| Good | 71% | 82 | 78.85% |
| Requires improvement / inadequate | 12% | 8 | 8.26% |
| **Pupil characteristics (whole school)** | | | |
|  |  | No. schools missing data, n/N (%) | Mean (SD) |
| % pupils eligible for FSM in the past 6 years | 25% | 0/109 (0.00%) | 38.40% (17.00) |
| % pupils with EAL | 21% | 0/109 (0.00%) | 8.77% (15.23) |
| % eligible pupils with SEND support | 12.9% | 0/109 (0.00%) | 16.01% (7.33) |
| Key Stage 2: % pupils reaching expected standard in reading, writing and maths, 2019 | 65% | 10/109 (9.17%) | 65.83% (0.13) |
| Key Stage 2: % pupils reaching higher standard, 2019 | 11% | 10/109 (9.17%) | 9.87% (0.06) |
|  |  |  |  |

^1^Note, this data is only available for primary and secondary schools combined, so we have omitted it.

Table 21: Missing Data

| Variable | Randomised n/N^1^ | Missing N (%) | Assessed at baseline n/N | Missing N (%) | Assessed at Endline n/N | Missing N (%) |
| --- | --- | --- | --- | --- | --- | --- |
| **Gender** | 3,658/3,658^2^ | 0 (0%) | 2,638/2,647 | 0 (0%) | 791/2,638^2^ | 1,847 (70.02%) |
| **Age in moths (baseline)** | 3,658/3,658^2^ | 0 (0%) | 2,638/2,647 | 0 (0%) | - | - |
| **Free School Meal Eligibility** | 1,054 / 3,649 | 0 (0%) | 666 / 2,390 | 0 (0%) | 227 / 753 | 0 (0%) |
| **IDACI** | 3,649 / 3,649 | 0 (0%) | 2,384 / 2,390 | 0 (0%) | 753 / 753 | 0 (0%) |
| **EAL** | 349 / 3,649 | 0 (0%) | 182 / 2,390 | 0 (0%) | 68 / 753 | 0 (0%) |
| **SEN** | 457 / 3,649 | 0 (0%) | 257 / 2,390 | 0 (0%) | 68 / 753 | 0 (0%) |
| **YARC^3^** |  |  |  |  |  |  |
| Sound Isolation | - | - | 2,389/2,638 | 249 (9%) | 781 / 2,638 | 1,857 (70.00%) |
| Sound Deletion | - | - | 2,389/2,638 | 249 (9%) | 781 / 2,638 | 1,857 (70.00%) |
| Letter Sound Knowledge | - | - | - | - | 791 / 2,638 | 1,847 (70.02%) |
| Early Word Recognition | - | - | - | - | 791 / 2,638 | 1,847 (70.02%) |
| **SDQ^4^** |  |  |  |  |  |  |
| Emotional Problems | - | - | - | - | 1,124 / 2,638 | 1,514 (57.39%) |
| Conduct Problems | - | - | - | - | 1,124 / 2,638 | 1,514 (57.39%) |
| Hyperactivity | - | - | - | - | 1,124 / 2,638 | 1,514 (57.39%) |
| Peer Problems | - | - | - | - | 1,124 / 2,638 | 1,514 (57.39%) |
| Prosocial | - | - | - | - | 1,124 / 2,638 | 1,514 (57.39%) |
| Total Difficulties | - | - | - | - | 1,124 / 2,638 | 1,514 (57.39%) |
|  |  |  |  |  |  |  |

**Notes**

^1^ n=3,658 is the total sample which includes all pupils in Reception year at the 109 participating schools. Due to limited testing time during the pre-intervention phase (September-October 2019), not all Reception classes could be assessed. In schools with multiple Reception classes, one class was randomly selected to complete the pre-intervention assessment, but all Reception pupils in that school were randomised. In the original analysis plan, we planned to assign the average score of the assessed class to pupils in the non-assessed class. However, due to the high level of attrition, we did not proceed with this planned imputation and instead conduced a complete case analysis.

^2^ n=9 pupils withdrew from the study following randomisation leaving a remaining sample of n=3,649.

^3^ Due to a limited testing window at baseline (September-October 2019), only two subscales of the YARC rather than all four were administered.

^4^ The SDQ was completed at endline only. In schools with multiple Reception classes, the class randomly selected to complete the baseline assessment was asked to complete the SDQ.

### **SI 7: Interaction Models – Treatment effects across sub-groups**

**Sub-group: Eligible for Free School Meals (FSM)**


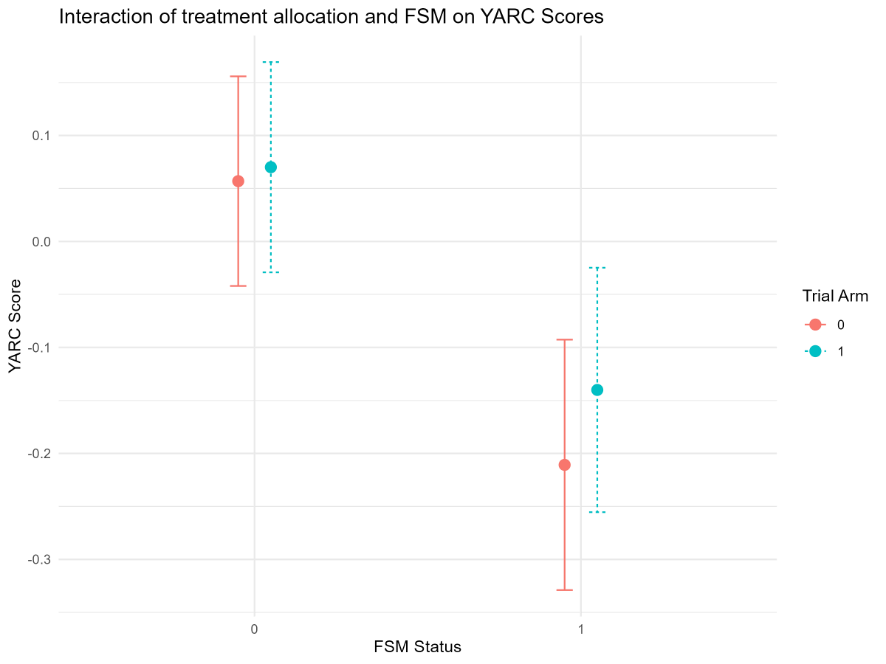


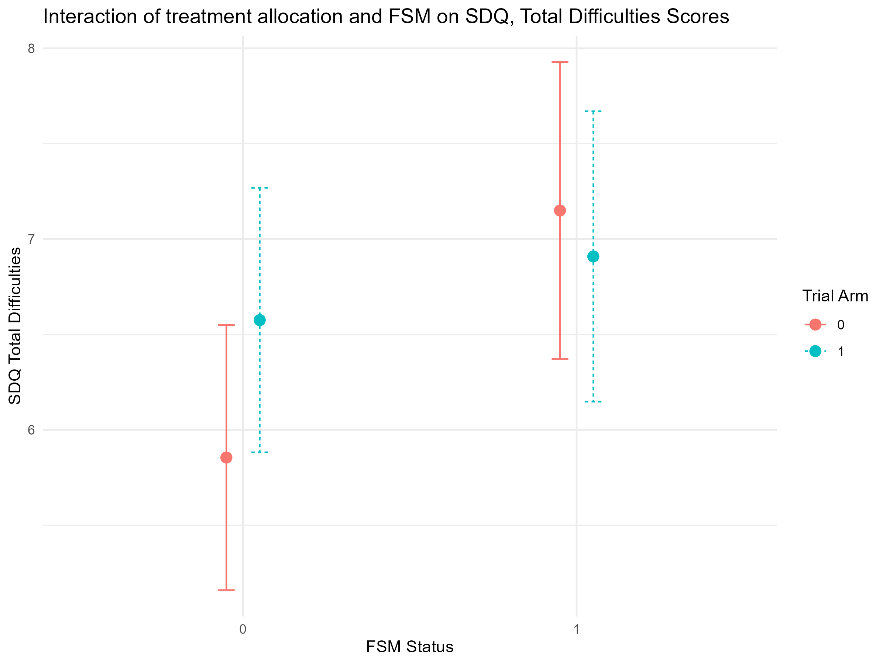


**Interaction model: Treatment × IDACI**

**
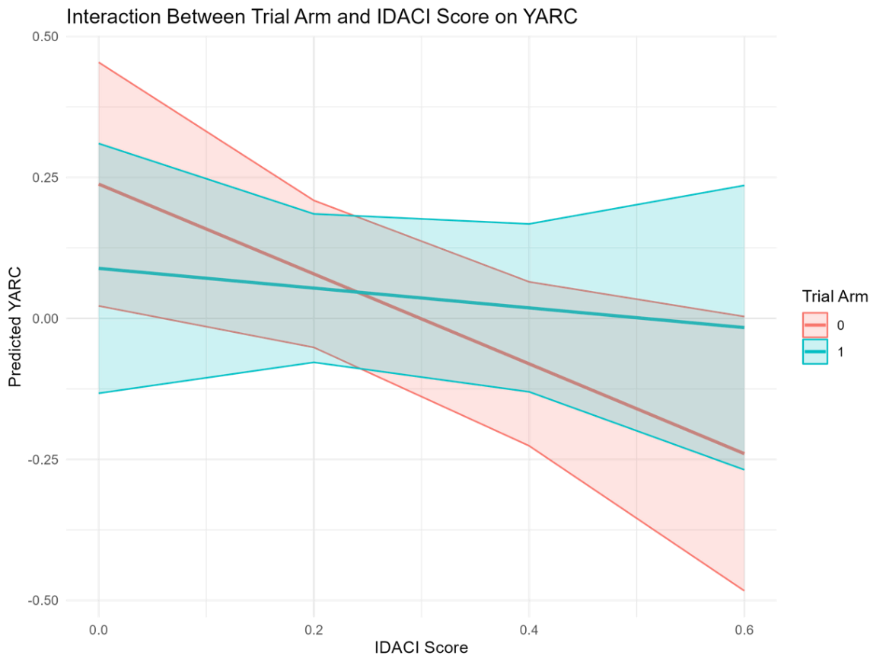
**

**
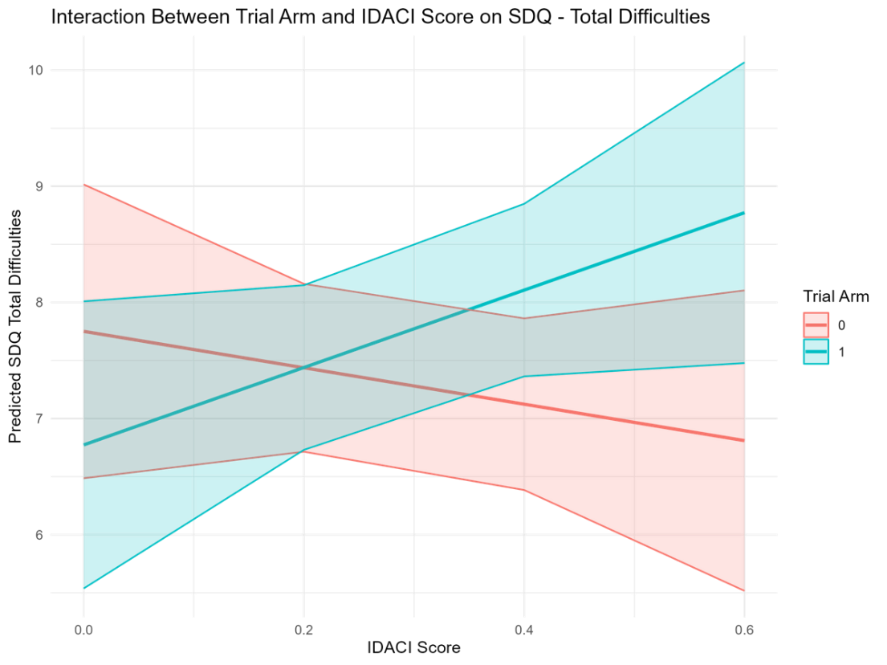
**

**Sub-group: Students with English as an Additional Language**

**
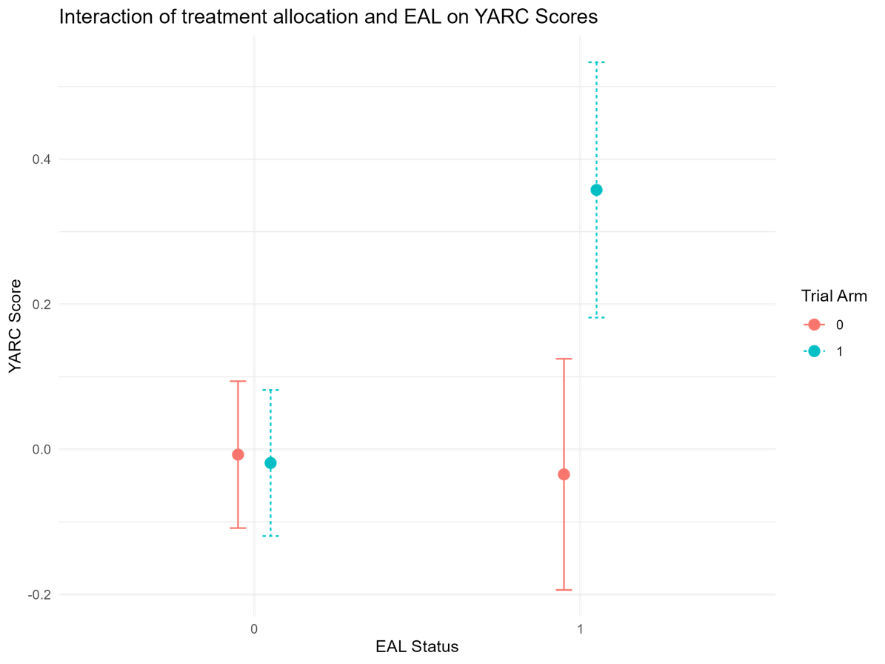
**

**
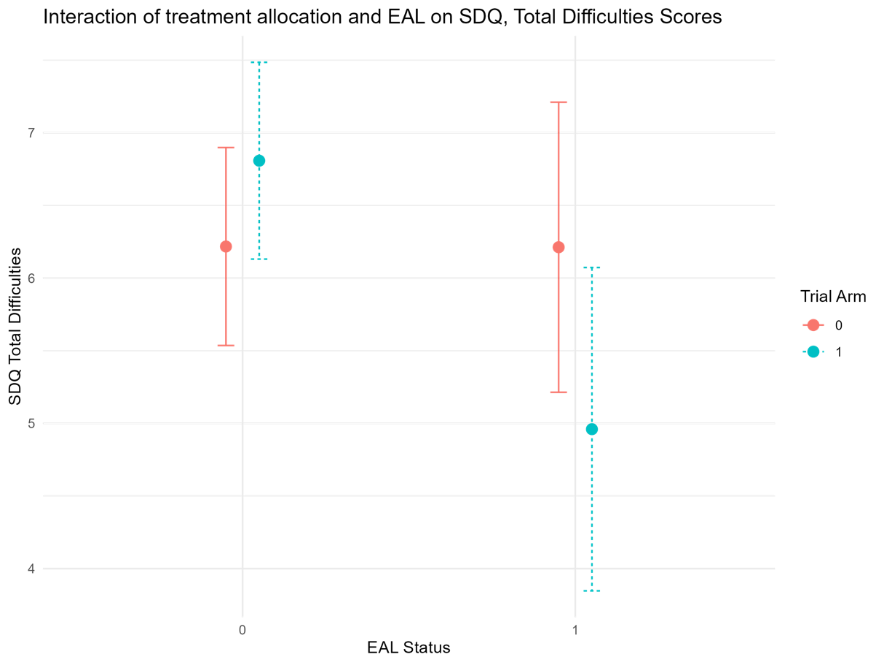
**

**Sub-group: Students with Special Educational Needs**

**
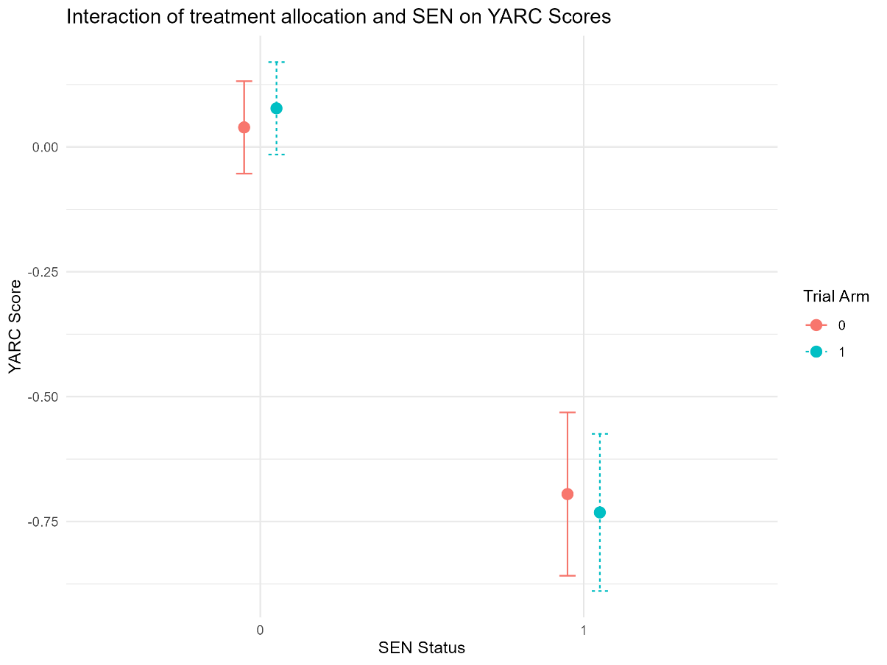
**

**
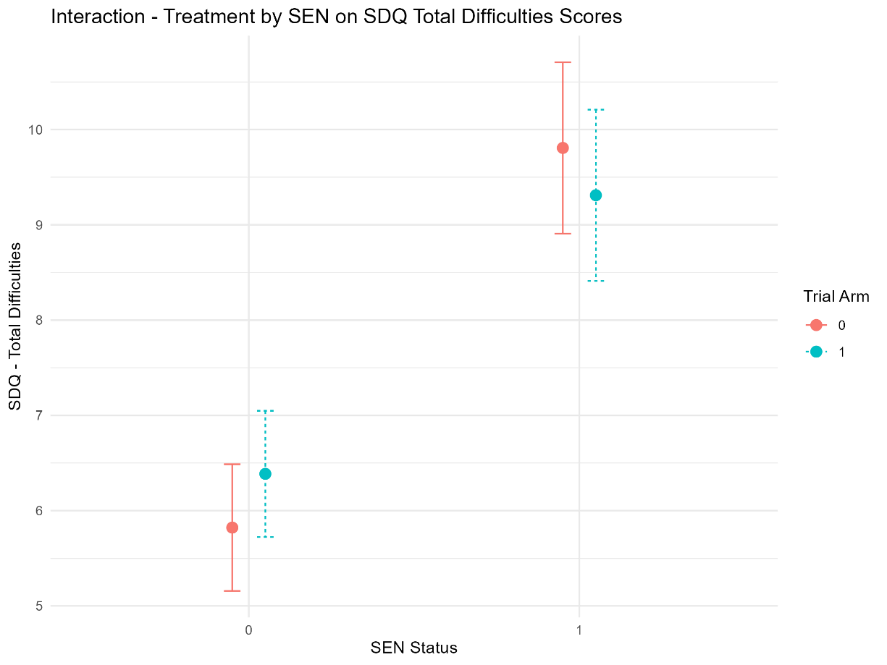
**

### **SI 8: Examining SDQ scores across IDACI quintiles**

Figure 4: Mean SDQ Total Difficulties Scores at different levels of IDACI


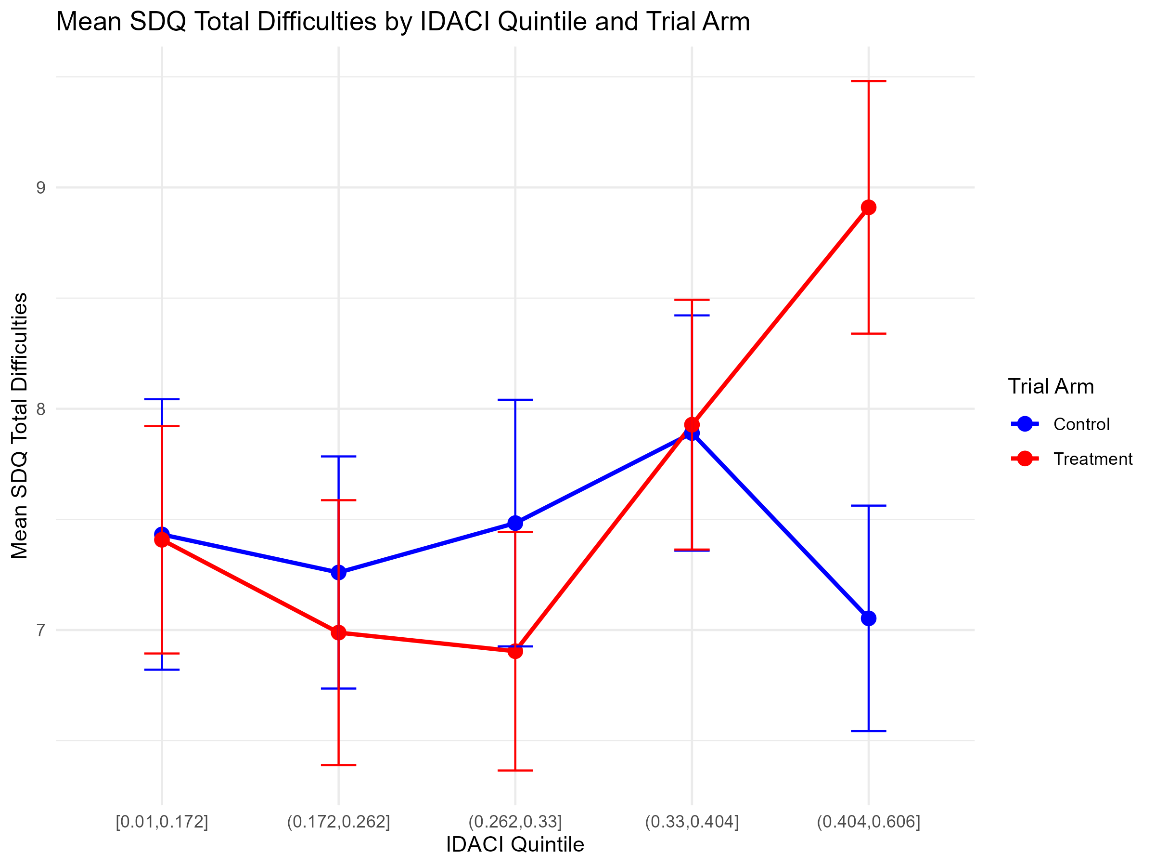


### **SI 9: Additional information on the Robustness Checks**

**Calculating Age in months at the point of randomization**

Student ages were calculated in months as of the end-of October 2019, the date of randomization. Since the dataset contained only year and month of birth (but not exact birth dates), each student’s date of birth was estimated by assuming birth occurred on the fifteenth day of the recorded birth month (e.g., a child born in June 2015 was assigned a birthdate of June 15, 2015). Age in months was then computed by taking the difference between the reference date (October 31, 2019) and the assigned birthdate, dividing by the average number of days in a month (30.44), and rounding to the nearest whole month. This approach ensures consistency with standard age calculation methods used in developmental psychology research.

This method follows standard procedures for estimating age when precise birth dates are unavailable and has been used in prior research on early childhood development (e.g., Author et al., Year). Sensitivity analyses confirmed that alternative assumptions (e.g., assigning mid-month birthdates) did not substantively change the results.

**Estimating Spillover Effects on the Primary Outcome (YARC Scores)**

Given the nature of the intervention—text messages sent to parents—it is plausible that spillover may have occurred if parents in the treatment group shared intervention content with those in the control group. To assess the potential impact of such spillover, we estimated how different levels of spillover (i.e., 10% and 20% of the control group receiving indirect exposure to the intervention) might affect the treatment effect size, using Hedges’ g as our primary measure.

*Approach to Estimating Spillover*

To quantify spillover, we first computed the proportion of treated pupils within each school and used this as a continuous measure of potential exposure among the control group. A mixed-effects model was fit to the full sample to estimate the main treatment effect on YARC scores, controlling for baseline reading ability (YARC pre-test score), child age, gender, and school-level clustering.

To simulate the impact of spillover, we adjusted the control group’s outcome scores by reassigning a proportion (10% and 20%) of control group children as effectively ‘treated’—reflecting the assumption that they were indirectly exposed to intervention content. The adjusted dataset was then analyzed using the same mixed-effects model as the main impact analysis, ensuring consistency in estimation.

*Results*

In the absence of spillover, the estimated treatment effect size (Hedges’ g) was 0.0200. When 10% of the control group was assumed to be indirectly exposed to the intervention, the effect size declined to 0.0136, and with 20% spillover, it further declined to 0.0072. These results suggest that even modest levels of spillover could attenuate the estimated treatment effect, potentially biasing results toward the null. Overall, while the intervention effect on YARC scores remained small, the spillover analysis suggests that diffusion of intervention materials among parents could partially explain the attenuated treatment effects observed in the main analysis. These findings highlight the need for careful consideration of spillover when designing and interpreting trials of parent-targeted interventions.

### **SI 10: False Discover Rate (FDR) adjusted p-values**

Table 22: Accounting for Multiple Comparisons - Raw and Adjusted p-values

| **Analysis** | | **N** | **Raw P-value** | **FDR Adjusted P-value** |
| --- | --- | --- | --- | --- |
| **Main impact analysis** | | | | |
| 1 | Impact YARC | 753 | 0.730 | 0.869 |
| 2 | Impact SDQ | 1,037 | 0.123 | 0.383 |
|  |  |  |  |  |
| **Sub-group: High Achievers** | | | | |
| 3 | Low achievers (YARC) | 181 | 0.331 | 0.618 |
| 4 | High achievers (YARC) | 188 | 0.787 | 0.869 |
| 5 | Treatment*low achievers (YARC) | 753 | 0.32 | 0.618 |
| 6 | Low achievers (SDQ) | 237 | 0.784 | 0.869 |
| 7 | High achievers (SDQ) | 283 | 0.190 | 0.443 |
| 8 | Treatment*low achievers (SDQ) | 1,037 | 0.516 | 0.803 |
|  |  |  |  |  |
| **Sub-group: Free School Meals (FSM)** | | | | |
| 9 | FSM Eligible (YARC) | 227 | 0.424 | 0.742 |
| 10 | FSM Non-Eligible (YARC) | 526 | 0.807 | 0.869 |
| 11 | Treatment*FSM (YARC) | 753 | 0.652 | 0.869 |
| 12 | FSM Eligible (SDQ) | 309 | 0.707 | 0.869 |
| 13 | FSM Non-Eligible (SDQ) | 727 | 0.054 | 0.288 |
| 14 | Treatment*FSM (SDQ) | 1,036 | 0.162 | 0.412 |
|  |  |  |  |  |
| **Sub-group: English as an Additional Language (EAL)** | | | | |
| 15 | EAL (YARC) | 68 | 0.045* | 0.288 |
| 16 | Non-EAL (YARC) | 345 | 0.864 | 0.896 |
| 17 | Treatment*EAL (YARC) | 753 | 0.047* | 0.288 |
| 18 | EAL (SDQ) | 81 | 0.072 | 0.288 |
| 19 | Non-EAL (SDQ) | 955 | 0.071 | 0.288 |
| 20 | Treatment*EAL (SDQ) | 1,036 | 0.119 | 0.383 |
|  |  |  |  |  |
| **Sub-group: Special Educational Needs (SEN)** | | | | |
| 21 | SEN (YARC) | 35 | 0.964 | 0.964 |
| 22 | Non-SEN (YARC) | 334 | 0.552 | 0.813 |
| 23 | Treatment*SEN (YARC) | 753 | 0.703 | 0.869 |
| 24 | SEN (SDQ) | 61 | 0.452 | 0.744 |
| 25 | Non-SEN (SDQ) | 459 | 0.070 | 0.288 |
| 26 | Treatment*SEN (SDQ) | 1,036 | 0.260 | 0.56 |
|  |  |  |  |  |
| **Sub-group: Neighborhood Disadvantage (IDACI)** | | | | |
| 27 | Treatment*IDACI (YARC) | 753 | 0.152 | 0.412 |
| 28 | Treatment*IDACI (SDQ) | 931 | 0.048* | 0.288 |
|  |  |  |  |  |

### **SI 11: Comparing SDQ results with and without baseline YARC**

Table 23: SDQ Total Difficulties without and without controlling for baseline YARC scores.

| **Outcome** | **Unadjusted means*** | | | | **Effect size** | | |
| --- | --- | --- | --- | --- | --- | --- | --- |
|  | *Control* | | *Treatment* | |  | | |
|  | *N* | *Mean (SD)* | *N* | *Mean (SD)* | *Total N* | *Hedge’s g (95% CI)* | *p-value* |
| **SDQ: Total Difficulties (Controlling for YARC at baseline)** | | | | | | | |
| Total Difficulties | 517 | 6.936  (5.534) | 520 | 7.035  (5.662) | 1,037 | 0.086 (-0.023, 0.196) | 0.123 |
| Internalizing | 517 | 2.512 (2.798) | 520 | 2.568 (2.737) | 1,037 | 0.067 (-0.046, 0.179) | 0.248 |
| Externalizing | 517 | 4.423 (4.004) | 520 | 4.467 (4.211) | 1,037 | 0.070 (-0.040, 0.182) | 0.208 |
| Prosocial | 517 | 7.218 (2.649) | 520 | 7.361 (2.523) | 1,037 | -0.019 (-0.124, 0.085) | 0.714 |
| **SDQ: Total Difficulties (Not controlling for YARC at baseline)** | | | | | | | |
| Total Difficulties | 517 | 6.669 (5.441) | 520 | 6.869 (5.606) | 1,037 | 0.045 (-0.067, 0.158) | 0.430 |
| Externalizing | 517 | 4.273 (3.932) | 520 | 4.356 (4.155) | 1,037 | 0.033 (-0.081, 0.147) | 0.572 |
| Internalizing | 517 | 2.397 (2.777) | 520 | 2.513 (2.702) | 1,037 | 0.043 (-0.071, 0.157) | 0.457 |
| Prosocial | 517 | 7.362 (2.566) | 520 | 7.423 (2.500) | 1,037 | 0.010 (-0.096, 0.115) | 0.858 |

Table 24: SDQ Total Difficulties among low and high achievers with and without controlling for baseline YARC scores.

|  | **Unadjusted means** | | | | **Effect size** | | |
| --- | --- | --- | --- | --- | --- | --- | --- |
|  | *Control* | | *Treatment* | |  |  |  |
|  | *N* | *Mean (SE)* | *N* | *Mean (SD)* | *Total N* | *Hedge’s g (95% CI)* | *p-value* |
| **SDQ: Total Difficulties (Controlling for YARC at baseline)** | | | | | | | |
| Low achievers (below median on baseline YARC) | 268 | 8.093 (5.528) | 237 | 8.228 (5.455) | 505 | 0.054 (-0.113, 0.220) | 0.537 |
| High achievers (above median on baseline YARC) | 249 | 5.137 (4.913) | 283 | 5.731 (5.485) | 532 | 0.135 (-0.020, 0.290) | 0.088 |
|  |  |  |  |  |  |  |  |
| **SDQ: Total Difficulties (Not controlling for YARC at baseline)** | | | | | | | |
| Low achievers (below median on baseline YARC) | 268 | 8.093 (5.528) | 237 | 8.228 (5.455) | 505 | 0.053 (-0.114, 0.220) | 0.536 |
| High achievers (above median on baseline YARC) | 249 | 5.137 (4.913) | 283 | 5.731 (5.485) | 532 | 0.110 (-0.046, 0.267) | 0.166 |

Table 25: Examining the interaction between Treatment and IDACI on SDQ Total Difficulties, with and without controlling for baseline YARC scores.

|  | **β** | **S. E** | **t-value** | **p-value** | **N** |
| --- | --- | --- | --- | --- | --- |
| **SDQ: Total Difficulties (Controlling for YARC at baseline)** | | | | | |
| Treatment | -0.977 | 0.796 | -1.227 | 0.220 | 931 |
| IDACI | -1.568 | 1.891 | -0.829 | 0.407 | 931 |
| Treatment × IDACI | 4.899 | 2.476 | 1.979 | 0.048* | 931 |
|  |  |  |  |  |  |
| **SDQ: Total Difficulties (Not controlling for YARC at baseline)** | | | | | |
| Treatment | -1.027 | 0.816 | -1.258 | 0.209 | 931 |
| IDACI | -0.921 | 1.923 | -0.479 | 0.632 | 931 |
| Treatment × IDACI | 4.484 | 2.536 | 1.768 | 0.077 | 931 |

Table 26: SDQ Total Difficulties among FSM-eligible and non-eligible students, with and without controlling for baseline YARC scores.

|  | **Unadjusted means** | | | | **Effect size** | | |
| --- | --- | --- | --- | --- | --- | --- | --- |
|  | *Control* | | *Treatment* | |  |  |  |
|  | *N* | *Mean (SE)* | *N* | *Mean (SD)* | *Total N* | *Hedge’s g (95% CI)* | *p-value* |
| **SDQ: Total Difficulties (Controlling for YARC at baseline)** | | | | | | | |
| FSM eligible | 147 | 7.605 (5.687) | 162 | 7.253 (5.510) | 309 | -0.041  (-0.256, 0.173) | 0.707 |
| FSM non-eligible | 369 | 6.287 (5.306) | 358 | 6.70 (5.648) | 727 | 0.130  (-0.002, 0.262) | 0.054 |
|  |  |  |  |  |  |  |  |
| **SDQ: Total Difficulties (Not controlling for YARC at baseline)** | | | | | | | |
| FSM eligible | 147 | 7.605 (5.687) | 162 | 7.253 (5.510) | 309 | -0.108  (-0.326, 0.109) | 0.329 |
| FSM non-eligible | 369 | 6.287 (5.306) | 358 | 6.696 (5.648) | 727 | 0.101  (-0.035, 0.236) | 0.146 |

Table 27: SDQ Total Difficulties among EAL and non-EAL students, with and without controlling for baseline YARC scores.

|  | **Unadjusted means** | | | | **Effect size** | | |
| --- | --- | --- | --- | --- | --- | --- | --- |
|  | *Control* | | *Treatment* | |  |  |  |
|  | *N* | *Mean (SE)* | *N* | *Mean (SD)* | *Total N* | *Hedge’s g (95% CI)* | *p-value* |
| **SDQ: Total Difficulties (Controlling for YARC at baseline)** | | | | | | | |
| EAL | 46 | 7.435 (5.445) | 35 | 6.029 (5.009) | 81 | -0.379  (-0.791, 0.033) | 0.072 |
| Non EAL | 470 | 6.587 (5.443) | 485 | 6.930 (5.647) | 955 | 0.106  (-0.009, 0.220) | 0.071 |
|  |  |  |  |  |  |  |  |
| **SDQ: Total Difficulties (Not controlling for YARC at baseline)** | | | | | | | |
| EAL | 46 | 7.435 (5.447) | 35 | 6.029  (5.009) | 81 | -0.328  (-0.758, 0.102) | 0.135 |
| Non EAL | 470 | 6.587 (5.444) | 485 | 6.930  (5.647) | 955 | 0.062  (-0.056, 0.180) | 0.301 |

Table 28: SDQ Total Difficulties among SEN and non-SEN students, with and without controlling for baseline YARC scores.

|  | **Unadjusted means** | | | | **Effect size** | | |
| --- | --- | --- | --- | --- | --- | --- | --- |
|  | *Control.* | | *Treatment* | |  |  |  |
|  | *N* | *Mean (SE)* | *N* | *Mean (SD)* | *Total N* | *Hedge’s g (95% CI)* | *p-value* |
| **SDQ: Total Difficulties (Controlling for YARC at baseline)** | | | | | | | |
| SEN | 63 | 11.190 (6.133) | 61 | 10.410 (5.841) | 124 | -0.137  (-0.492, 0.219) | 0.452 |
| Non-SEN | 454 | 6.042 (5.033) | 459 | 6.399 (5.409) | 913 | 0.107  (-0.009, 0.223) | 0.070 |
|  |  |  |  |  |  |  |  |
| **SDQ: Total Difficulties (Not controlling for YARC at baseline)** | | | | | | | |
| SEN | 63 | 11.190 (6.133) | 61 | 10.410 (5.841) | 124 | -0.134 (-0.488, 0.220) | 0.458 |
| Non-SEN | 454 | 6.042 (5.033) | 459 | 6.399 (5.409) | 913 | 0.062 (-0.056, 0.181) | 0.304 |
|  |  |  |  |  |  |  |  |

### **SI 12: YARC baseline data**


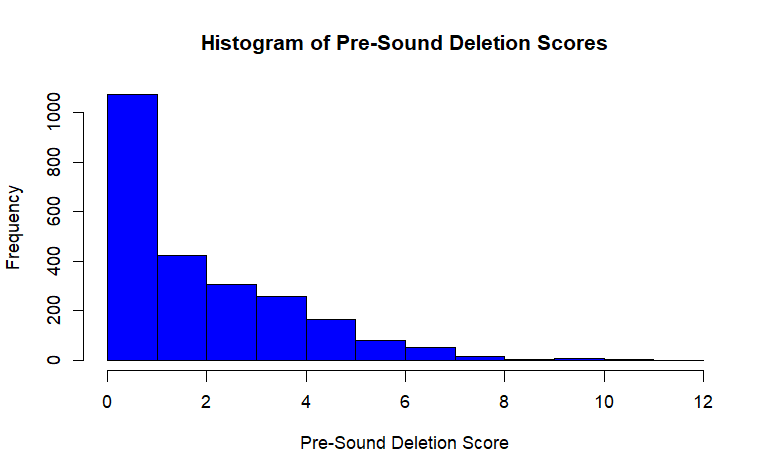


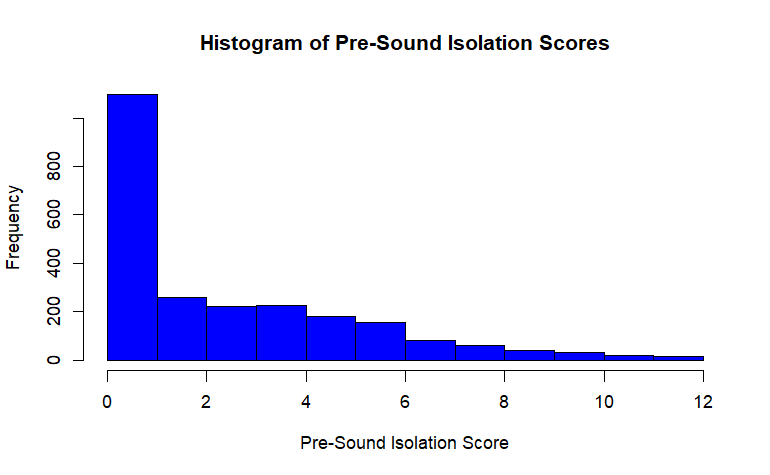

Supplement: Supplementary file 1 [file edu0000973_Supplemental_Materials.docx]
